# Awaiting discovery: How biases in faunistic surveys hinder conservation in mountain protected areas—A case study from Romania’s oldest national park

**DOI:** 10.1371/journal.pone.0319871

**Published:** 2025-04-01

**Authors:** Dan Cogălniceanu, Dorel Ruşti, Marius Skolka, Florina Stănescu, Sabina E. Vlad, Teodora L. Tănase, Laurenţiu Rozylowicz

**Affiliations:** 1 Research Center of the Natural Sciences Department, Ovidius University of Constanţa, Constanţa, Romania; 2 Asociația Harta Verde România, Bistrița-Năsăud, Romania; 3 Asociația Chelonia România, Bucharest, Romania; 4 Center for Morphological and Genetic Studies of Malignant Pathology (CEDMOG), Black Sea Institute for Development and Security Studies (BSIDSS), Ovidius University of Constanţa, Constanţa, Romania; 5 Center for Environmental Research, Bucharest, Romania; Instituto Federal de Educacao Ciencia e Tecnologia Goiano - Campus Urutai, BRAZIL

## Abstract

It is virtually impossible to compile a complete list of species and map their distributions in any protected area; nevertheless, a near complete inventory is vital for an adequate management plan. We use Retezat National Park (Romania) as a case study to assess the bias in faunistic inventories over the past 70 years. Retezat National Park is one of the most studied in Eastern Europe since it shelters some of the last patches of virgin old-growth forest in Europe. We reviewed the scientific literature published since the early 1900s dealing with faunistic surveys and retrieved the occurrence records available on GBIF for the study area. We identified a total of 4374 animal species belonging to 2113 genera, 494 families, 99 orders, 23 classes, and nine phyla. The number of publications started to accumulate after 1979, when the park became a Biosphere Reserve, reached a peak in the early 2000 and severely decreased during the last decade, highlighting a decline in the researchers’ interest in faunistic surveys and, possibly, a delay between data collection and their publication. GBIF-mediated data made a small contribution, most records (60%) being collected in 2019. The bias analyses included only the distribution records available in the scientific literature, since they were already validated through peer-review. The number of publications and background of experts involved influenced the taxonomic coverage and inventory completeness. We found a strong spatial bias in terms of inventory coverage, with diversity hotspots located near roads and research facilities. Our study provides a roadmap for cost-effective future faunistic studies by prioritizing conservation efforts towards the most understudied areas and taxa within the park.

## Introduction

Biodiversity loss is one of the main challenges of our time, as species and habitats are vanishing at an unprecedented rate, on a trajectory towards a Sixth Mass Extinction [1]. As concerns over biodiversity declines are growing, the amount, complexity, and rate at which new environmental and ecological data are generated have also increased exponentially. This surge in data availability fuels the research and discovery engine driving biodiversity conservation efforts [2] and has advanced our knowledge of biodiversity sensitivity to environmental changes. It also helps support the implementation of evidence-based policies and practices that can help mitigate the worst outcomes.

Despite the tremendous development of biodiversity informatics [e.g., 3,4], much published data remains unused and is not easily available to decision-makers and the research community. While biodiversity assessments play a crucial role in successful conservation planning, many protected areas still lack comprehensive biodiversity inventories, particularly for important species‐rich taxa (e.g., invertebrates) that deliver essential ecosystem services (e.g., pollination, nutrient cycling) and serve as valuable biological indicators [5]. Species inventories and mapping of species occurrences are the first step in achieving consistent, integrated conservation policies [6].

Biodiversity is unevenly distributed around the world, and is often highly concentrated in relatively few small areas, usually at the Tropics. Because of this distribution pattern, conservation efforts have to be directed towards the regions/taxa/ecosystems where they are most needed. A variety of methods have been proposed to support prioritization in biodiversity conservation, from simple richness ranking [7] to identifying hotspots [8], and designating Important Biodiversity Areas [9] or Key Biodiversity Areas [10]. Such methods rely on data obtained through species inventories. This is why incomplete and biased inventories pose a real problem, often leading to unsuccessful conservation measures and wasted resources. Biases and gaps in biodiversity surveys have been shown to reduce the management efficiency in protected areas despite large territories being designated as protected in the last decade [11–13].

Mountains cover a large part of Earth’s terrestrial surface and host a higher proportion of biodiversity than expected by area [14], representing an estimated one-third of terrestrial species diversity [15]. Mountain ecosystems are exposed to multiple threats, from climate change to direct habitat degradation by human activity. These have drastic biological consequences in the most vulnerable mountain ecosystems, posing a direct threat to human well-being and health, and ultimately to the global life-support systems [16]. To properly mitigate and manage these threats, adequate, unbiased biodiversity inventories and surveys are required.

The Romanian Carpathians are considered a biodiversity hotspot in Europe, with limited human development, large and compact virgin forests, and biodiversity-rich grasslands [17]. They are better managed compared to other regions in Romania, with over 60% of the Southern Carpathians included in protected areas; 22 out of the 27 national and nature parks are located in the Carpathians [11,18]. Despite their protected status, few studies have attempted to provide a synthesis of species richness and distribution, and even fewer have discussed the changes in biodiversity patterns and causal factors, such as land-use changes or human development. Furthermore, limited data on Carpathians’ biodiversity contribute to legally-binding management plans that fail to include evidence-based conservation actions, thus further limiting the efficacy of protected areas [12,17].

Perhaps the most studied protected area in the Romanian Carpathians is Retezat National Park (RNP), the first national park designated as such in 1935. The area within the park boundaries has been subjected to multiple management practices over time as a consequence of changes in property type and legislation. Historically, it faced various human activities, such as lumber exploitation, hunting, fish introductions, pastoralism/grazing, and tourism; some of these practices continue to the current date [19]. The Romanian Academy facilitated research conducted in this national park through the establishment of two research stations (i.e., Gura Zlata and Gemenele) nearby and within Gemenele Scientific Reserve (GSR). The high conservation value and rich biodiversity of this reserve have triggered multiple, large-scale, targeted studies. As such, the first two monographs regarding the diversity and distribution of plants [20] and caddisflies [21] were published in the 1950s. Five years after the inclusion of the park in the World Network of Biosphere Reserves through UNESCO’s Man and Biosphere Program, a monograph covering various aspects of the fauna and flora of RNP was published [22]. Two other monographs were published in the 1990s, dealing with ecological aspects [23] and the insect fauna [24] of RNP. A monograph concerning the geomorphological diversity of the park was published in 2000 [25]. The most recent monograph, published in 2022, concerns the vascular plant communities in RNP [26]. Apart from these, there are hundreds of scientific papers devoted to species diversity and distribution in various ecosystems of the park. Still, a large proportion of this information is difficult to access.

We analyze the biases and completeness of faunistic surveys in RNP based on research published over the past 70 years as a case study to highlight the risks and vulnerabilities faced by conservation managers in biodiversity-rich mountain protected areas. More specifically, we (i) provide a unitary and updated inventory of the animal species richness in RNP, (ii) identify gaps in knowledge, taxonomic and spatial biases, and finally (iii) provide guidelines to support future conservation and management activities.

## Materials and methods

### Study area

**Fig 1:**
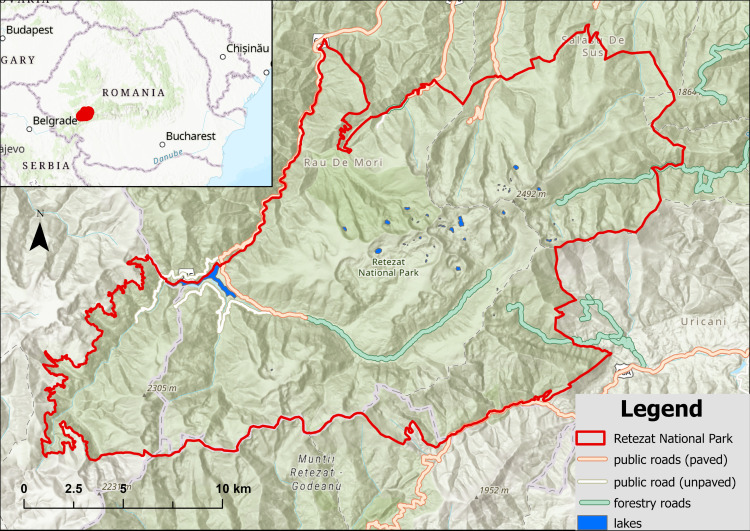
Overview of Retezat National Park location in Romania. The inset image shows the park’s location (in red) in Romania and the Southern Carpathians. The red border corresponds to Retezat National Park. This figure was created using the basemap World Topographic Map [27].

Retezat National Park is located in the Retezat Mountains, part of the Southern Carpathians, in Romania (Fig 1). It covers a total area of 38047 ha, of which 4350 ha was designated in 1955 as a strictly protected area (Gemenele Scientific Reserve) with restricted public access [26]. The altitude in RNP ranges from 600 m to 2509 m. The lower parts of the park shelter deep, narrow valleys, while the higher parts consist of glacial plateaus. The main geological features consist of Danubian metamorphic rocks dominated by slightly metamorphosed crystalline schists; the glacial relief allows lakes to form in the deeper parts of the moraines [28]. Fifty-eight permanent glacial lakes and an almost equal number of temporary lakes are recorded at altitudes between 1700 m and 2300 m [29]. Runoff varies with altitude, from 14.3 L/s/km^2^ between 600 and 800 m to 28 L/s/km^2^ between 1600 to 1800 m, and up to 36.6 L/s/km^2^ at altitudes higher than 2200 m. The highest monthly runoff is typically recorded in May [28]. The climate is continental, moderately cold and humid (ET and Dfc according to Köppen classification [30]), with yearly average temperatures between 6°C in the valleys and −2°C in the alpine areas; the annual rainfall varies between 900 to 1300 mm, reaching higher values below the tree line [31]. Plant diversity is high, with 1152 plant species reported, representing about one third of the total for Romania [26]. The park shelters 19 endemic species of insects (nine Lepidoptera, six Plecoptera, four Trichoptera) and 57 endemic plant species and subspecies [26]. The largest single area of pristine mixed forest in Europe can be found within the lower-altitude parts of the scientific reserve [32]. The park was included in the Natura 2000 network of protected areas in 2007; it was withdrawn from the World Network of Biosphere Reserves in 2021.

### Literature review

We performed an exhaustive literature search dealing with faunistic studies in RNP, informal searches of papers collected over the years, and contacts with experts in the field. A significant proportion of species lists was compiled from “Fauna of Romania”, the national scientific series dedicated to specific animal taxa, first published in 1951, with 307 volumes to date. For recent publications, we carried out an extensive literature search on the Web of Science and Google Scholar. We used “Retezat” as a keyword, and then we manually selected the publications of interest. The reference sections of these publications were further screened for other potentially relevant articles that were not identified during the initial search. We considered only the scientific publications from 1948 onward since older publications lack precision in geographic location and provide outdated taxonomic classifications. Furthermore, valid data from older references are most often included in subsequent publications. We made an exception regarding the time limit for Lepidoptera by considering the work of Diószeghy [33,34], which already included 1091 out of the 1372 species inventoried to the present (79.5%).

Inconsistencies and errors in naming the surveyed taxa and taxonomic classification were the most challenging when collecting data from old sources. Typographic errors were relatively common, especially in older publications. Since some authors have published repeatedly on a particular taxon using different species names, we followed the taxonomic classification of the most recent publications, including, when appropriate, species that were omitted in a later publication. The taxonomy was checked and updated according to GBIF Backbone Taxonomy [4], and when higher taxa classification was not available, we also referred to the Catalogue of Life (COL) [3]. The number of species in higher taxa categories for selected invertebrate taxa was also retrieved from COL. The sampling methods used in the analyzed papers varied depending on the target taxa and the type of habitat, but most publications did not provide this information; therefore, we considered that most of the data were obtained opportunistically. For comparison purposes, we also retrieved the animal species records for Retezat National Park from GBIF [35] (a link to the query conditions and dataset is provided with the reference). We excluded the entries where the “Species” column was empty (i.e., 110 entries out of 3015 retrieved by the query).

### Data analysis

While we recognize the value of public online databases, we included only data records from the scientific literature in the following analyses since experts have validated these through a peer-review process. We used EstimateS v. 9.1 [36] to compute species accumulation curves (SACs) to assess inventory completeness for the taxonomic groups where sufficient data were available. A SAC is a plot of the cumulative number of species inventories within a particular area as a function of some measure of the effort required to find them [37]. We constructed SACs for the best-studied groups using the number of publications per decade as a measure of the sampling effort. Since the data extracted from the different publications was not homogenous, we could not apply any extrapolation methods, especially estimators of species richness. We assessed the completeness of faunistic inventories by visual inspection of the SACs. Thus, a SAC that is near a plateau indicates that most species have been inventoried; when still increasing, a SAC shows that more species await discovery, and therefore, additional surveys are necessary.

Whenever the precise geographical location was provided, this was recorded for further analyses. However, not all occurrences reported from RNP have known locations associated. Some papers provide only a list of species, sometimes mentioning the sampling sites but without providing details of species encountered at each site, referring to broad locations (e.g., Râul Mare Valley) or simply referring to general toponyms like “Retezat mountains”. After excluding records without precise locations, we obtained a dataset of 6124 occurrence records within the perimeter of RNP (S1 Database).

To assess survey bias at the national park scale, we used the Getis-Ord Gi * spatial statistic for counts of occurrences aggregated at 1 km^2^ grid cells. This allowed us to identify clusters of occurrence records (i.e., grid cells with values higher than expected by random chance within a specified searching distance) [38]. The distance threshold for the aggregation patterns was set to 3625.7 m, to include the neighboring eight grid cells of each cell of interest [39]. For every grid cell, the Getis Ord Gi * test returns a Z-score describing spatial clusters of high (hotspots)/ low (coldspot) sampling effort or non-significant aggregation. All spatial analyses were performed using ArcGIS Pro 3.1.0 [39].

## Results

### General overview

**Fig 2:**
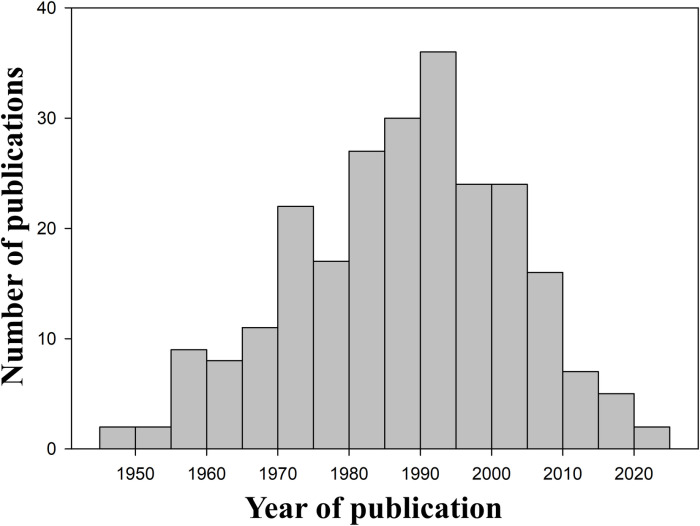
Dynamics of the scientific publications regarding the fauna of Retezat National Park. The number of scientific papers published between 1948 and 2023 reporting faunistic occurrences from RNP.

The literature review included 256 publications (S2 File), mostly scientific papers (73%) and books/ book chapters (27%). Except for two papers published in 1930 and 1935, all other studies were published between 1948 and 2023. We observed a peak in the number of publications between 1990 and 1995, followed by a rapid decrease during the last two decades (Fig 2).

**Fig 3:**
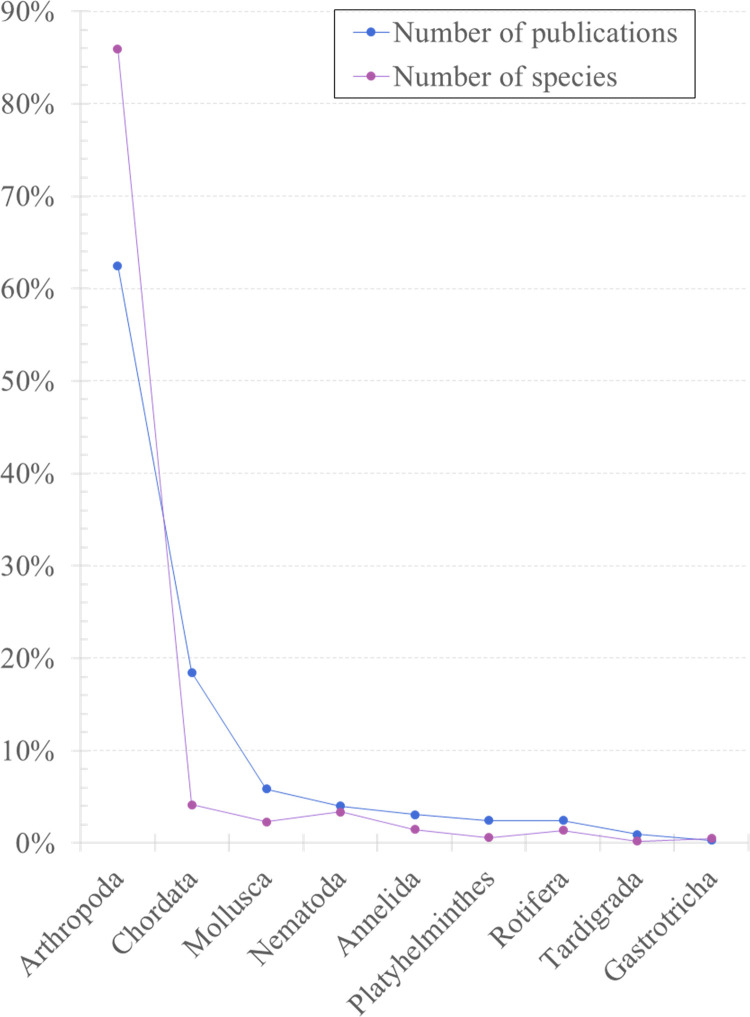
Animal species richness reported by the scientific literature for Retezat National Park, and the corresponding number of published papers. The number of animal species recorded in RNP closely follows the number of published scientific papers. Values are presented as a percentage of the total number of publications and total number of species, respectively.

Overall, the number of scientific publications was significantly correlated to the number of species (Spearman’s *rho* =  0.633, *P* <  0.001; SigmaPlot 11.0 [40]) recorded in RNP. The arthropods were the most studied taxa from RNP (Fig 3), the scientific studies being focused on four insect orders: Diptera (20%, 51 publications), Coleoptera (10%, 25 publications), Lepidoptera (8%, 21 publications), and Hymenoptera (6%, 16 publications). Vertebrates were the second most studied group, but the data available was mostly limited to species lists for each class, and did not allow for additional analyses.

The scientific literature contributed with records of 4301 species and 6124 georeferenced occurrences, while GBIF contributed with 334 species and 2905 georeferenced occurrences (between 1929 and 2023). GBIF occurrence records were biased towards birds (69%), with most of the data collected in 2019. GBIF added 73 species not previously recorded in the scientific literature for RNP (59% insects, 11% other invertebrates, 30% birds). Based on both sources combined, the current animal species richness for RNP sums up to 4374 species, grouped in nine phyla, the highest proportion corresponding to insects, with 3211 species (73%) (S3 Table, S4 Database).

### Sampling bias

**Fig 4:**
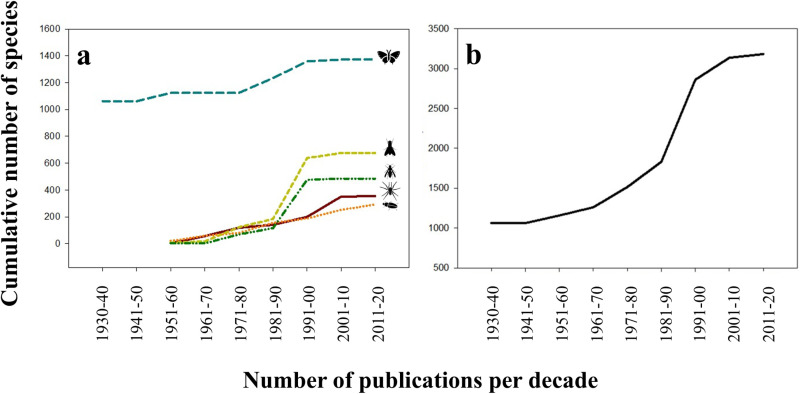
Assessment of inventory completeness in five of the most studied taxa from Retezat National Park. (a) The cumulative number of invertebrate species of the most abundant five higher taxa: Class Arachnida (dark red, continuous line) and Class Insecta with the Orders Lepidoptera (blue interrupted line), Diptera (yellow interrupted line), Hymenoptera (green interrupted line) and Coleoptera (orange dotted line). As an exception, we added occurrences since 1930 only for Lepidoptera since these were available in the seminal papers by Diószeghy [33,34]. (b) The total cumulative number of invertebrate species for the most abundant five higher taxa.

**Fig 5:**
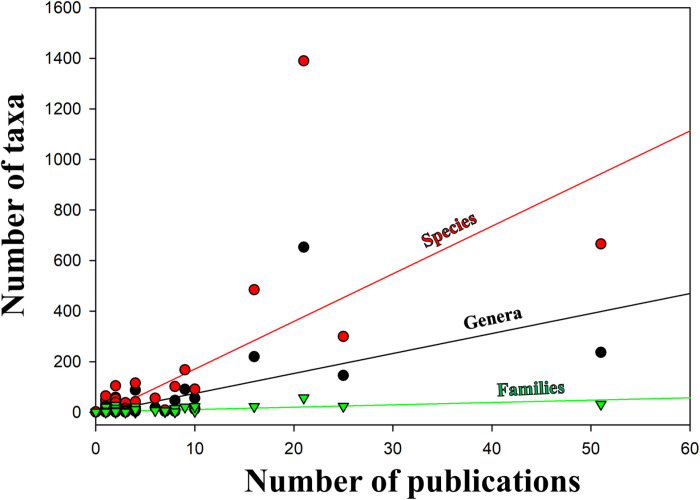
The relationship between the number of publications and taxa richness in Retezat National Park. The positive linear relationship between the number of publications and taxa richness is presented at three taxonomic levels: species (red line, red dots; *R*^*2*^ =  0.51), genera (black line, black dots; *R*^*2*^ =  0.45), and families (green line, green triangles; *R*^*2*^ =  0.43). The graph was created based on the data from the S3 Table.

We were able to compute SACs for five of the most studied invertebrate taxa: Arachnida and Insecta - Coleoptera, Diptera, Hymenoptera, Lepidoptera). SACs indicated that the inventory is almost complete in four of these (i.e., Arachnida and Insecta – Diptera, Hymenoptera, Lepidoptera), but most species are yet to be recorded for Coleoptera (Fig 4a). The number of invertebrate species inventoried by specialists showed a steep increase in the 1980s and 1990s and reached a plateau during the last decade (Fig 4b), reflecting the publishing pattern. This is consistent with the positive relationship between the number of publications and species richness at multiple taxonomic levels (Fig 5).

**Fig 6:**
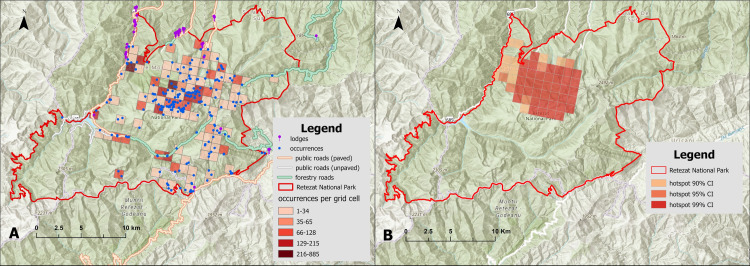
Spatial bias in faunistic surveys in Retezat National Park. (a) Sampling locations of the faunistic studies within Retezat National Park corresponding to a grid of 1 km^2^ cells. (b) Hotspots of sampling at 1 km^2^ cells; the red to orange gradient corresponds to Getis Ord Gi * Z-scores estimated with different confidence thresholds (i.e., light orange =  90%, dark orange =  95%, and red =  99% confidence). This figure was created using the basemap World Topographic Map [27].

**Fig 7:**
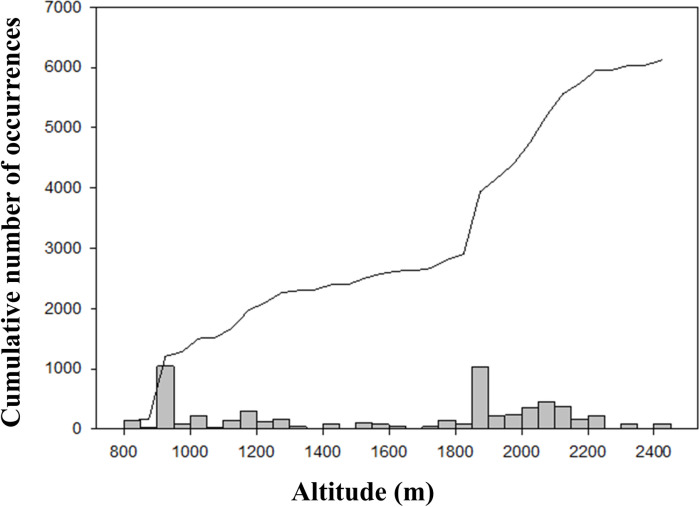
The cumulative number of species occurrences according to altitude in Retezat National Park. The number of occurrences peaks around two altitudes corresponding to the research stations (Gura Zlata and Casa Laborator Gemenele). The line represents the cumulative number of occurrences.

The spatial analysis revealed a biased sampling in RNP (Fig 6a), with only 58 out of 460 1 km^2^ grid cells overlapping RNP having more than one occurrence record (12.6%). The mean number of occurrences was 12.8 per grid cell (SD =  64.44), while the maximum number of occurrences per grid cell was recorded at Casa Laborator Gemenele (885 occurrences) and Gura Zlata (864 occurrences). A number of 356 grid cells (77%) had no occurrences. The hotspots are located near camping and accommodation facilities like Zănoaga, Gemenele, Bucura, and Gura Zlata. In contrast, the grid cells that were not significantly sampled are located in the western, southern, and eastern parts of RNP (Fig 6b). The distribution of species occurrences across altitude revealed two peaks corresponding to the research facilities: Gura Zlata (850 m) and Casa Laborator Gemenele (1770 m) (Fig 7).

## Discussion

Our study highlights that although Retezat National Park is the best-studied mountain protected area in Romania, based on the number of publications, the knowledge of animal species richness is still biased, both in terms of taxonomic and spatial coverage. We showed that vast areas of the park remain understudied (i.e., the western, southern, and eastern parts of RNP), and only a few areas were properly sampled (see Fig 6). Even under these circumstances, we found a high faunistic richness when compared to other similar national parks in Romania, confirming its high conservation value. This is valid even if most taxonomic groups are undersampled and the sampling during the over 70 years was strongly biased towards the iconic research infrastructure of the Romanian Academy (i.e., Casa Laborator Gemenele and Gura Zlata).

**Fig 8:**
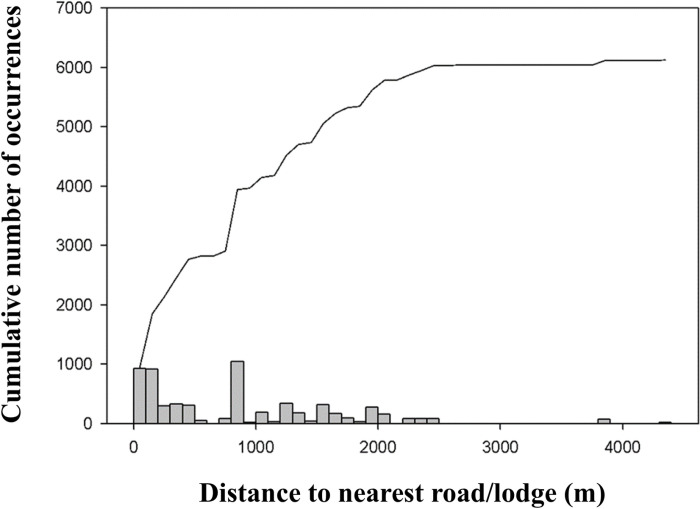
Relationship between the distance to the nearest road/lodge and cumulative number of species occurrence records in Retezat National Park. The line represents the cumulative number of species occurrences.

The spatial and taxonomic biases in faunistic surveys from RNP are important but similar to most historical inventories [41]. For example, the spatial bias observed in our study is partly explained by the distance to the nearest infrastructure (e.g., road, shelter) (Fig 8) and thus by difficulties in access and lack of accommodation facilities. This situation is consistent with results obtained in other parts of the world. For example, Ferreira et al. [42] showed that the availability of accommodations for researchers was the only variable influencing the likelihood of conducting arthropod inventories in a protected area from Brazil. Falcón-Brindis et al. [43] found similar patterns of sampling bias for moths (Insecta, Erebidae) in the Chiapas region of Mexico, highlighting the gap between the process of designating protected areas, their efficiency, and the basic and representative knowledge of what is/ needs to be protected. Our study contributes to the growing evidence pointing out that research must be expanded beyond the easily-accessible areas, close to roads or research stations. To make this possible, increased logistic support from protected areas administrators or other stakeholders (e.g., local authorities) is of paramount importance, especially in terms of providing accommodation facilities in remote and otherwise inaccessible areas.

**Fig 9:**
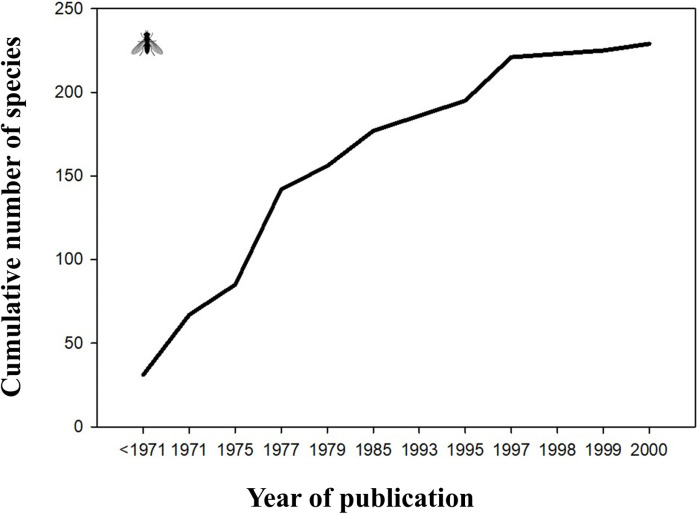
Species richness of Syrphidae (order Diptera) from Retezat National Park. The figure depicts the cumulative number of species of Syrphidae reported from RNP, based on the work of Vladimir Brădescu between 1971 and 2000 (see S2 File for the complete list of publications). Before his studies started, only 31 species were known from the park, reaching 229 species by 2000.

We found a disproportionate importance that a small number of experts had in the spatial sampling of underrepresented taxa (e.g., Vladimir Brădescu for Syrphidae, Diptera, or László Diószeghy for Lepidoptera; S2 File). Thus, the higher proportion of known species in some groups is the result of the work of a few dedicated individuals. This shows that the data available is not necessarily representative of the actual biodiversity richness in RNP, but an indicator of the researchers’ interest in certain taxa or study areas, the so-called “botanist effect” [44]. For example, Connor and Simberloff [45] showed that the number of field trips for conducting botanical surveys to each of the Galapagos islands was a better predictor of species number than any physical characteristics of the respective islands. Similarly, we show that the differences in species richness between the different higher-taxa categories in RNP are most likely a reflection of the uneven sampling effort. In RNP, the family Syrphidae (Insecta, Diptera) was intensively studied by the same expert (i.e., Vladimir Brădescu) for three decades, an effort that produced 14 publications on this group (Fig 9, S2 File). A similar situation can be observed in butterflies and moths (Insecta, Lepidoptera), the best-studied group among all invertebrate categories, with 1390 species identified in RNP. This is due to the fact that Lepidoptera is one of the emblematic groups of insects and an attraction for both researchers and naturalists; besides, Retezat Mountains were less affected by grazing compared to the surrounding massifs, thus providing extended areas of relatively well-conserved habitats for this group. The first exhaustive synthesis regarding the Lepidoptera of RNP was done by László Diószeghy, who published an annotated list of over 1000 species of micro- and macro-Lepidoptera in 1930 [33]. An updated synthesis was published much later, in 1997, by László Rákosy [24]. Despite their attractiveness, a coherent and sustained inventory and monitoring program for moths and butterflies was never established in RNP. This further increases the inventory bias among Lepidoptera groups, which is already influenced by size and charisma. The species richness of Lepidoptera currently known from RNP amounts to 34% of the total recorded by Rákosy and Goia [46] for the whole country (i.e., 4073 species). As expected, this proportion is biased towards butterflies (Rhopalocera; 64%), while Microlepidoptera, a less attractive group, is underrepresented (25%).

A complete census of species in any given area is rarely feasible, except for highly visible, sessile, and well-studied taxa [47]. Biodiversity assessment is, therefore, usually based on samples, but the species count depends on sampling effort. We used SACs to overcome the challenge of comparing the heterogeneous datasets among inventories (i.e., based on different sampling methodologies and efforts) [48]. These allowed us to estimate the completeness of species inventory in the higher taxa and provide an estimate of species richness for the curves that approached a plateau. The overall low number of studies for most taxa limited the applicability of SACs to five invertebrate higher taxa (i.e., Arachnida and Insecta – Coleoptera, Diptera, Hymenoptera, Lepidoptera). The knowledge gaps in terms of taxonomic and spatial coverage were high, with Coleoptera, the most abundant order of insects worldwide, being understudied. For example, the first inventory of invertebrates from the Retezat Mountains that we could identify was based on collections made during 1898-1899 by Szilády [49]. It reported 834 species of arthropods, mostly insects (90%), followed by Arachnida, Chilopoda, and Crustacea. Since the sampling was not focused on a specific group and the sampling methods were not presented, the proportion of the species richness of higher insect taxa differed from our checklist. A comparison of the species richness in selected insect taxa reported from Retezat National Park, according to the inventory done by Szilády [49], the present study (i.e., data from the scientific literature and GBIF), and at a global scale (Catalogue of Life) [3] reveals a high taxonomic bias in the faunistic surveys after 1900 (Table 1). The highest discrepancies can be observed in Coleoptera, significantly understudied after 1948 (negative bias), and Lepidoptera, intensively studied, starting with the seminal papers of Diószeghy [33,34] (positive bias).

**Table 1:** Species richness in selected insect orders reported from Retezat National Park reveals strong taxonomic bias. Species richness in selected insect orders reported from Retezat National Park, according to the inventory done by Szilády [49], the present study (i.e., data from the scientific literature and GBIF), and at a global scale (Catalogue of Life) [3]. A strong negative bias can be observed in Coleoptera after 1906. Conversely, a strong positive bias can be observed towards Lepidoptera.

Order	Szilády (1906)		Present study		Global	
	Number	%	Number	%	Number	%
**Orthoptera**	29	4	50	2	28111	3
**Coleoptera**	299	41	300	10	316269	36
**Lepidoptera**	57	8	1390	46	149969	17
**Diptera**	112	15	666	22	165144	19
**Hymenoptera**	137	19	485	16	118145	14
**Hemiptera**	93	13	116	4	93749	11
**Total**	**727**	**100**	**3007**	**100>**	**871387**	**100>**

While our results are mostly centered around invertebrates, where the data available allowed for more complex analyses, we consider that the findings of this study can be extrapolated to other groups of taxa and regions, even in megadiverse regions, with high proportions of endemisms and undescribed species. For example, a recent study [50] showed that sampling completeness in vertebrates decreases with higher species richness and lower country-level per capita GDP; thus, spatial sampling biases are more likely to occur in megadiverse regions because of difficult access imposed by the scarcity of resources (i.e., financial, equipment, knowledge/experts), infrastructure or harsh environment (natural and/or political). Designing cost-effective inventories remains a challenging activity since funding is limited and the rate of species discovery declines rapidly as sampling effort or area increases [51]. While increasing the number and frequency of inventories and overall research effort is valuable for many reasons, this would probably lead to more surveys of already sampled areas, as the lack of accessibility and research infrastructure promotes close distances between new and existing sampling sites [52]. To sustain an acceptable level of cost-effectiveness, we recommend to (1) prioritize sampling efforts towards previously undersampled groups or areas, and (2) start using data from other approaches, such as occupancy [53], when making management decisions. These general recommendations are suitable for most study areas or taxa and address the major pitfalls in faunistic surveys identified in our study (i.e., spatial and taxonomic biases). We provide the foundation for designing effective faunistic surveys, by highlighting the undersampled taxa and areas within the park, as a case study, which should be of special interest to wildlife managers. One particular finding that arose from this study is related to the taxonomic bias and the critical contributions of few dedicated experts to specific groups of taxa. As such, investment in maintaining an adequate pool of research expertise should be a national priority for governments and education/research institutions, especially in the Anthropocene, the era marked by massive biodiversity loss. Acquiring high-quality data on biodiversity has never been more crucial; biases in data sampling lead to biased decisions and potentially catastrophic consequences.

## Supporting information

S1 Database Georeferenced faunistic occurrence records reported from Retezat National Park. The database includes occurrences extracted from the scientific literature that provided sufficient information regarding location. These data were used for the spatial analyses (see also Fig 6). In this version, species names were not corrected or updated.
(XLSX)

S2 File Scientific literature regarding the fauna from Retezat National Park (RNP), Romania.
List of references revised to extract faunistic occurrence data reported from RNP.
(DOCX)

S3 Table Synthetic overview of the faunistic diversity reported from Retezat National Park (RNP), based on data from the scientific literature and GBIF.
The table presents a summary of the animal diversity reported from RNP, according to the scientific literature and GBIF [35]. The taxonomy follows GBIF Backbone Taxonomy [4] and the Catalogue of Life (COL) [3]. NA =  “not assigned”. Taxa are ordered alphabetically. The numbers in parentheses represent the total number of unique items of each category.
(DOCX)

S4 Database Animal species richness reported from Retezat National Park (RNP), according to the scientific literature and GBIF. The database contains all species recorded from RNP, according to the scientific literature and GBIF [35]. The taxonomy follows GBIF Backbone Taxonomy [4] and the Catalogue of Life (COL) [3]. NA =  “not assigned”.
(XLSX)

## References

[1] Cowie RH, Bouchet P, Fontaine B. The sixth mass extinction: Fact, fiction or speculation? Biol Rev Camb Philos Soc. 2022;97(2):640–63. doi: 10.1111/brv.12816 35014169 PMC9786292

[2] MacFadyen S, Allsopp N, Altwegg R, Archibald S, Botha J, Bradshaw K, et al. Drowning in data, thirsty for information and starved for understanding: A biodiversity information hub for cooperative environmental monitoring in south Africa. Biol Conserv. 2022;109736.

[3] Bánki O, Roskov Y, Döring M, Ower G, Hernández Robles DR, Plata Corredor CA, et al. Catalogue of Life Checklist. 2023 Oct 16 [Cited 2024 June 15]. Available from: doi: 10.48580/df7lv

[4] GBIF Secretariat GBIF Backbone Taxonomy. Checklist dataset. 2023 Nov 11 [Cited 2024 June 15]. Available from: doi: 10.15468/39omei

[5] Girardello M, Martellos S, Pardo A, Bertolino S. Gaps in biodiversity occurrence information may hamper the achievement of international biodiversity targets: Insights from a cross-taxon analysis. Environ Conserv J. 2018;45(4):370–7.

[6] Borzée A. 13-step framework for better integration of streamlined conservation research. Integr Conserv. 2023;2(3):156–64.

[7] Jenkins CN, Pimm SL, Joppa LN. Global patterns of terrestrial vertebrate diversity and conservation. Proc Natl Acad Sci U S A. 2013;110(28):E2602–10. doi: 10.1073/pnas.1302251110 23803854 PMC3710798

[8] Myers N, Mittermeier RA, Mittermeier CG, da Fonseca GA, Kent J. Biodiversity hotspots for conservation priorities. Nature. 2000;403(6772):853–8. doi: 10.1038/35002501 10706275

[9] Donald PF, Fishpool LD, Ajagbe A, Bennun LA, Bunting G, Burfield IJ, et al. Important bird and biodiversity areas (ibas): The development and characteristics of a global inventory of key sites for biodiversity. Bird Conserv Int. 2019;29(2):177–98. doi: 10.1017/S095927091900001X

[10] Kullberg P, Di Minin E, Moilanen A. Using key biodiversity areas to guide effective expansion of the global protected area network. Glob Ecol Conserv. 2019;20:e00768.

[11] Manolache S, Ciocănea CM, Rozylowicz L, Niță A. Natura 2000 in Romania – A decade of governance challenges. Eur J Geogr. 2017;8(2):24–34.

[12] Miu IV, Rozylowicz L, Popescu VD, Anastasiu P. Identification of areas of very high biodiversity value to achieve the EU biodiversity strategy for 2030 key commitments. PeerJ. 2020;8:e10067. doi: 10.7717/peerj.10067 33062449 PMC7532765

[13] Geue JC, Rotter PJ, Gross C, Benkő Z, Kovács I, Fântână C, et al. Limited reciprocal surrogacy of bird and habitat diversity and inconsistencies in their representation in Romanian protected areas. PLoS One. 2022;17(2):e0251950. doi: 10.1371/journal.pone.0251950 35148309 PMC8836316

[14] Körner C. Mountain biodiversity, its causes and function. Ambio. 2004;33:11–7. 15575177

[15] Körner C, Paulsen J, Spehn EM. A definition of mountains and their bioclimatic belts for global comparisons of biodiversity data. Alpine Botany. 2011;121:73–8.

[16] Schmeller DS, Urbach D, Bates K, Catalan J, Cogălniceanu D, Fisher MC, et al. Scientists’ warning of threats to mountains. Sci Total Environ. 2022;853:158611. doi: 10.1016/j.scitotenv.2022.158611 36087665

[17] Stanciu E, Iojă IC, Tintarean M, Pop MP. Romania. In: Tucker G, Editor. Nature Conservation in Europe: Approaches and Lessons. Cambridge: Cambridge University Press. 2023. pp. 534–54.

[18] Iojă CI, Pătroescu M, Rozylowicz L, Popescu VD, Vergheleț M, Zotta MI. The efficacy of Romania’s protected areas network in conserving biodiversity. Biol Conserv. 2010;143:2468–76. 10.1016/j.biocon.2010.06.013

[19] Rose NL, Cogălniceanu D, Appleby PG, Brancelj A, Camarero L, Fernández P. Atmospheric contamination and ecological changes inferred from the sediment record of Lacul Negru in the Retezat National Park, Romania. Adv Limnol. 2009;62:319–350.

[20] Nyarady El. Flora şi vegetaţia Munţilor Retezat. Bucharest: Editura Academiei RPR; 1958.

[21] Botoşăneanu L. Cercetări asupra Trichopterelor din Masivul Retezat și Munţii Banatului. Bucharest: Editura Academiei RPR; 1959.

[22] Pascu S, Negruţiu E, Puia I, Boşcaiu N, Popovici I. Recherches écologiques dans le Parc National de Retezat. Bucharest: Editura Academiei RPR; 1984.

[23] Popovici I. Parcul Naţional Retezat. Studii ecologice. Brașov: West Side Computers; 1993.

[24] Rákosy L. Entomofauna Parcurilor Naţionale Retezat şi Valea Cernei. Cluj-Napoca: Societatea Lepidopterologică Română; 1997.

[25] Urdea P. Munţii Retezat. Studiu geomorfologic. Bucharest: Editura Academiei Române; 2000.

[26] Coldea G, Cristea V. The vascular plant communities of the Retezat National Park (Southern Carpathians). Cham: Springer Nature; 2022.

[27] ESRI World Topographic Map v2. 2024 [cited 2024 Sep 15]. Available from: https://www.arcgis.com/home/item.html?id=7dc6cea0b1764a1f9af2e679f642f0f5

[28] Schreiber W, Sorocovschi V. Munții Retezat. Condiții fitogeografice. Parcul Național Retezat. Studii ecologice. Brașov: West Side Computers; 1993:8–12.

[29] Pişotă I. Lacurile glaciare din Carpații Meridionali. Bucharest: Editura Academiei Române; 1971.

[30] Ontel I, Cheval S, Irimescu A, Boldeanu G, Amihaesei VA, Mihailescu D, et al. Assessing the recent trends of land degradation and desertification in Romania using remote sensing indicators. Remote Sens. 2023;15(19):4842. doi: 10.3390/rs15194842

[31] Fărcaş I, Sorocovschi V. Clima Munților Retezat. Parcul Național Retezat. Studii ecologice. Brașov: West Side Computers; 1993:13–20.

[32] Veen P, Fanta J, Raev I, Biriş IA, de Smidt J, Maes B. Virgin forests in Romania and Bulgaria: Results of two national inventory projects and their implications for protection. Biodivers Conserv. 2010;19(7):1805–19.

[33] Diószeghy L. Die lepidopterenfauna des retyezatgebirges [The lepidoptera fauna of the Retezat Mountains]. Verh Mitt Siebenb Ver Naturwiss Hermannstadt. 1930;79–80.

[34] Diószeghy L. Die lepidopterenfauna des retyezatgebirges. Nachtrag I [The lepidoptera fauna of the Retezat Mountains. Addendum I]. Verh Mitt Siebenb Ver Naturwiss Hermannstadt. 1935;83–84.

[35] Global Biodiversity. Occurrence download. 2024. doi: 10.15468/dl.nnkgs5

[36] Colwell RK. EstimateS: Statistical estimation of species richness and shared species from samples. 2019. http://purl.oclc.org/estimates

[37] Colwell RK, Coddington JA. Estimating terrestrial biodiversity through extrapolation. Philos Trans R Soc Lond B Biol Sci. 1994;345(1311):101–18. doi: 10.1098/rstb.1994.0091 7972351

[38] Ord JK, Getis A. Local spatial autocorrelation statistics: Distributional issues and an application. Geogr Anal. 1995;27:286–306.

[39] ESRI Optimized Hot Spot Analysis (Spatial Statistics); 2023. Available from: https://pro.arcgis.com/en/pro-app/3.1/tool-reference/spatial-statistics/optimized-hot-spot-analysis.htm

[40] SigmaPlot for Windows Version 11.0, Stystat Software Inc. Copyright 2008.

[41] Hortal J, Jiménez‐Valverde A, Gómez JF, Lobo JM, Baselga A. Historical bias in biodiversity inventories affects the observed environmental niche of the species. Oikos. 2008;117(6):847–58.

[42] Ferreira GP, Antunes AZ, Campagnoli ML, Christianini AV. Sleeping accommodations for researchers increase the likelihood of biodiversity inventories in protected areas. Integr Conserv. 2023;2(1):62–8. 10.1002/inc3.19

[43] Falcón-Brindis A, León-Cortés JL, Montañez-Reyna M. How effective are conservation areas to preserve biodiversity in Mexico? Perspect Ecol Conserv. 2021;19(4):399–410. 10.1016/j.pecon.2021.07.007

[44] Moerman DE, Estabrook GF. The botanist effect: Counties with maximal species richness tend to be home to universities and botanists. J Biogeogr. 2006;33(11):1969–74.

[45] Connor EF, Simberloff D. Species number and compositional similarity of the Galapagos flora and avifauna. Ecol Monogr. 1978;48(2):219–48.

[46] Rákosy L, Goia M. Lepidopterele din România: lista sistematică și distribuție [The Lepidoptera of Romania: a Distribution Checklist]. Cluj-Napoca: Presa Universitară Clujeană; 2021.

[47] Beck J, Schwanghart W. Comparing measures of species diversity from incomplete inventories: An update. Methods Ecol Evol. 2010;1(1):38–44.

[48] Moreno CE, Halffter G. Assessing the completeness of bat biodiversity inventories using species accumulation curves. J Appl Ecol. 2000;37(1):149–58.

[49] Szilády Z. Az izeltábúak függélzes elterjedése a Ryetezát faunájából vett példákol. [On the Vertical Distribution of Insects Using Examples from the Fauna of the Retezat]. Múzeumi Füzetek, Klausenburg. 1906;1:159–95.

[50] Šmíd J. Geographic and taxonomic biases in the vertebrate tree of life. J Biogeogr. 2022;49(12):2120–9. 10.1111/jbi.14491

[51] Keating KA, Quinn JF, Ivie MA, Ivie LL. Estimating the effectiveness of further sampling in species inventories. Ecol Appl. 1998;8(4):1239–49.

[52] Carvalho RL, Resende AF, Barlow J, França FM, Moura MR, Maciel R, et al. Pervasive gaps in amazonian ecological research. Curr Biol. 2023;33(16):3495–3504.e4. doi: 10.1016/j.cub.2023.06.077 37473761

[53] Rozylowicz L, Popescu VD, Mirea MD, Manolache S, Miu IV, Niță A, et al. Wildpop: An interactive tool for estimating occupancy and abundance of wildlife populations. Carpathian J Earth Environ Sci. 2024;19(2):321–8. 10.26471/cjees/2024/019/302

