# Supplementary material for: Awaiting discovery: How biases in faunistic surveys hinder conservation in mountain protected areas—A case study from Romania’s oldest national park
Source: PLoS One. 2025 Apr 1;20(4):e0319871. doi: 10.1371/journal.pone.0319871 (PMC11961002; doi:10.1371/journal.pone.0319871)
Supplement: S2 File — List of references revised to extract faunistic occurrence data reported from RNP. (DOCX) [file pone.0319871.s002.docx]

**S2 File.** **Scientific literature regarding the fauna from Retezat National Park (RNP), Romania.** List of references revised to extract faunistic occurrence data reported from RNP.

1. Albu P 1974 Dinamica chironomidelor capturate într-o capcană cu lumină la Gura Zlata (Retezat). Sargetia 10: 129–136.
2. Almasan H, Soran V, Borcea M, Munteanu D, Coldea G 1986 Consideratii privind structura trofica a biomurilor din Muntii Retezat. Ocrotirea naturii si a mediului inconjurator 30: 13–24.
3. Avram S, Dumitrescu D 1969 Contribuţii la cunoaşterea răspândirii geografice şi a ecologiei opilionidelor cavernicole endogee şi epigee din Romania. Lucrarile Institutului de Speologie “Emil Racoviţă” 8: 99–145.
4. Avram S, Dancau D, Serban E 1964 Cercetari speologice in bazinul Cernei. Nota I. Lucrarile Institutului de Speologie Emil Racovita 3: 131–199.
5. Balog A 1999 Contribuția entomologilor maghiari la cunoașterea faunei de coleoptere din Transilvania de la început până la 1913 I Buletinul informativ al Societăţii Lepidopterologice Române 10 (1-4): 159-165
6. Bănăduc D 2006 The Râul Mare River (Retezat Mountains Romania) fish fauna Transylvanian Review of Systematical and Ecological Research 3: 117-124
7. Banarescu P 1964 Pisces. Osteichthyes Fauna RPR: Editura Academiei RPR
8. Banarescu P Erhan-Dinca E 1976 Contributii la cunoasterea zonarii biologice a unor piraie din Parcul National Retezat Sargetia 11-12: 221--233
9. Bechet I 1985 Psocopteres (Insecta Psocoptera) du Parc national de Retezat Studia Unviersitaria Babes-Bolyai Biologia 30: 51-54
10. Benedek A 2006. Small mammals from Retezat National Park (Romania). Transylvanian Review of Systematical and Ecological Research 3: 139-146
11. Benedek, A. M., Sîrbu, I., Lazăr, A. 2021 Responses of small mammals to habitat characteristics in Southern Carpathian forests. Scientific reports 11(1): 1-13
12. Benedek, A.M., Soricu, M., Druga, M 2004 Preliminary data regarding the fauna of small mammals (ord. Insectivora and ord. Rodentia) from the Retezat Mountains and the surrounding areas. Muzeul Bruckenthal-Studii si Comunicari, St. Nat. 29: 221-230
13. Botnariuc, N., Orghidan, T 1953 Crustacea. Phyllopoda. Fauna R.P.R. 4, fasc. 2: Editura Academiei R.P.R.
14. Botnariuc, N., Tatole, V. 1997 Consideratii asupra Chironomidofaunei Masivului Retezat. Ocrotirea naturii si a mediului inconjurator 41: 23-33
15. Botoc, M 1984 Chalcidoides du Parc national de Retezat. Recherches ecologiques dans le Parc National de Retezat : 239-241 Academia R.S.R., Cluj Napoca
16. Botosaneanu, L 1959 Cercetari asupra Trichopterelor din Masivul Retezat si Muntii Banatului. Editura Academiei RPR
17. Botosaneanu, L., Schneider, E.A. 1978 Trichopterele (Ord. Trichoptera) in colectiile Muzeului de Istorie Naturala din Sibiu. Muzeul Bruckental-Studii si Comunicari, St. Nat. 22: 307-326
18. Brădescu, V. 1971 Cercetari dipterologice (Syrphidae) in Parcul national Retezat. Ocrotirea naturii 15: 35-47
19. Brădescu, V. 1976 Consideratii zoogeografice si ecologice privind fauna Sirfidelor (Diptera) din Parcul National Retezat . Sargetia 12: 297-300
20. Brădescu, V. 1977 Noi cercetari dipterologice (Syrphidae) in Parcul national Retezat. Ocrotirea naturii si a mediului inconjurator 21: 33-38
21. Brădescu, V. 1979 Syrphides nouveaux dans la faune de Roumanie (Diptera, Syrphidae). Travaux Musee d'Histoire Naturelle "Grigore Antipa" 20: 293-295
22. Brădescu, V 1985 Date noi privind fauna dipterologica (Syrphidae) din Parcul national Retezat. Ocrotirea naturii si a mediului inconjurator 29: 57-58
23. Brădescu, V 1987 Nouvelles raretes dipterologiques dans la faune de Roumanie (Diptera, Syrphidae) . Travaux Musee d'Histoire Naturelle "Grigore Antipa" 29: 185-187
24. Brădescu, V 1993. Date noi privind fauna sirfidologica a Parcului national Retezat (Diptera, Syrphidae). Ocrotirea naturii si a mediului inconjurator 37: 49-50
25. Brădescu, V 1993. *Orthonevra shusteri* sp. N. et deux especes signalees pour la premiere fois dans la faune de Roumanie (Diptera, Syrphidae). Travaux Musee d'Histoire Naturelle "Grigore Antipa" 33: 7-10
26. Brădescu, V 1996. Noi date dipterologice (Syrphidae) pentru fauna Romaniei obtinute in anul 1995. Travaux Musee d'Histoire Naturelle "Grigore Antipa" 36: 291-294
27. Brădescu, V 1997 Donnees nouvelles concernant la faune des Syrphides (Diptera, Syrphidae) de Roumanie. Travaux Musee d'Histoire Naturelle "Grigore Antipa" 39: 183-186
28. Brădescu, V 1997 Interferente ecologice in entomofauna parcurilor nationale Retezat si Valea Cernei (Diptera: Syrphidae). Entomofauna parcurilor nationale Retezat si Valea Cernei: 229-242 Societatea Lepidopterologica Romana, Cluj Napoca
29. Brădescu, V 1998 Nota privind noi semnalari de Sirfide (Diptera) in Romania. Travaux Musee d'Histoire Naturelle "Grigore Antipa" 40: 367-370
30. Brădescu, V 1999 Species of *Cheilosia* (Diptera: Syrphidae) mentioned for the first time in Romanian fauna and in the National Park of Retezat Mountains. Travaux Musee d'Histoire Naturelle "Grigore Antipa" 41: 367-371
31. Brădescu, V 2000 Doua specii de Sirfide (Diptera) semnalate pentru prima data in Romania. Travaux Musee d'Histoire Naturelle "Grigore Antipa" 42: 165-166
32. Burnaz, S 1993 Catalogul colectiei de lepidoptere a muzeului judetean Hunedoara-Deva. Sargetia 14-15: 157-302
33. Burnaz, S 2002 Fauna de lepidoptere diurne (Ord. Lepidoptera, S. ord. Rhopalocera) a judeţului Hunedoara, România. Consideraţii ecologice, biologice şi zoogeografice. Buletinul informativ al Societăţii Lepidopterologice Române 13(1-4): 41-66
34. Burnaz, S., Konig, F 1984 Lepidopteres alpins-subalpins et boreo-alpins du Parc national de Retezat dans la collection du Musee du departement de Hunedoara-Deva. Recherches ecologiques dans le Parc National de Retezat : 231-238 Academia R.S.R., Cluj Napoca
35. Cameron, R. A., Pokryszko, B. M., Horsák, M., Sirbu, I., & Gheoca, V. 2011 Forest snail faunas from Transylvania (Romania) and their relationship to the faunas of Central and Northern Europe. Biological Journal of the Linnean Society 104: 471-479
36. Cameron, R. A., Pokryszko, B. M., Horsák, M., Sîrbu, I., Gheoca, V. 2011 Forest snail faunas from Transylvania (Romania) and their relationship to the faunas of Central and Northern Europe. Biological Journal of the Linnean Society 104(2): 471-479
37. Cantoreanu, M.1992. Structura comunitatilor de cicadide (Homoptera, Auchenorrhyncha). Parcul National Retezat -Studii Ecologice: 251-253. West Side Computers, Brasov
38. Capuse, I., Kovacs, A. 1987 Catalogul colectiei de lepidoptere "Laszlo Dioszeghy" de la Muzeul Judetean Covasna, Sfintu Gheorghe. Institutul de Speologie "Emil Racovitza", Bucuresti
39. Carausu, S., Dobreanu, E., Manolache, C. 1955 Crustacea. Amphipoda. Fauna R.P.R. 4, fasc. 4: Editura Academiei R.P.R.
40. Ceianu, I. 1989 Contributions to the knowledge of some families of diptera Acapyptrata in Romania. Travaux Musee d'Histoire Naturelle "Grigore Antipa" 30: 21-36
41. Ceianu, I. 1992 Contributions to the knowledge of superfamily Empidoidea (Diptera) in Romania. I.. Travaux Musee d'Histoire Naturelle "Grigore Antipa" 32: 17-48
42. Ceianu, I. 1998 Familia Phaeomyiidae (Diptera: Acalyptrata) in Romania. Bul. Inf. Soc. Lepid. Rom 9: 133-135
43. Ceianu, I. 1998b Contribution to the knowledge of the genus *Thricops* (Diptera: Muscidae) in Romania with description of a new species. Entomol. Rom. 3: 137-144
44. Ceianu, I., Martinek, V., Ghizdavu, L. 1991 Contribution to the knowledge of families Lauxaniidae and Heleomyzidae (Diptera, Acalyptrata) in Romania. Travaux Musee d'Histoire Naturelle "Grigore Antipa" 31: 85-112
45. Ceuca, T. 1984 Diplopodes du Parc national de Retezat. Recherches ecologiques dans le Parc National de Retezat: 197-200 Academia R.S.R., Cluj Napoca
46. Cirdei, F., Bulimar, F. 1965 Insecta. Odonata. Fauna RPR 7, fasc.5: Editura Academiei RPR
47. Ciubuc, C.1993. Checklist of Romanian Trichoptera (Insecta). Travaux Musee d'Histoire Naturelle "Grigore Antipa" 33: 11-147
48. Ciubuc, C. 2020 Carabidae din România / Carabidae from Romania: Pim. Iaşi
49. Cîmpean, M., Gerecke, R. 2006 Water mites (Acari, Hydrachnidia) from the Retezat National Park (Romania). Transylvanian Review of Systematical and Ecological Research 3: 69-78
50. Cobzaru, I. 2006 Contributions to the study of rodents in the Retezat National Park (Romania). Transylvanian Review of Systematical and Ecological Research 3: 125-138
51. Cogălniceanu, D. 1993. Studiul morfologic si morfometric al unor populatii de *Triturus alpestris* (Amphibia, Caudata) din Muntii Retezat (Carpatii Meridionali). Sargetia: 331-340
52. Cogălniceanu, D., Ghira, I., Ardeleanu, A. 2001 Spatial distribution of herpetofauna in the Retezat National Park (Romania). Biota 2: 9-16
53. Cogălniceanu, D., Tudorancea, M., Preda, E., Gâldean, N. 2009 Evaluating diversity of chironomid (Insecta: Diptera) communities in alpine lakes, Retezat National Park (Romania). Advances in Limnology 62: 191-213
54. Constantineanu, I., Constantineanu, R., Lungu-Constantineanu, C. 2003 New and rare exochinae (Hymenoptera: Ichneumonidae, Exochinae) for the Romanian fauna. Entomol. Rom. 7: 89-96
55. Constantineanu, R., Constantineanu, I. 1997 Ihneumonide (Hymenoptera, Ichneumonidae) din parcurile nationale Retezat si Valea Cernei. Entomofauna parcurilor nationale Retezat si Valea Cernei: 153-211 Societatea Lepidopterologica Romana, Cluj Napoca
56. Corocleanu, I. 1977 Alopiinele (Mollusca Gastropoda Pulmonata, fam. Clausiliidae) in colectiile Muzeului de Istorie naturala din Sibiu. Muzeul Bruckental-Studii si Comunicari, St. nat. 21.
57. Crișan, A. 2009 Cercetari asupra crizomelidelor (Coleoptera, Chrysomelidae) din aria Câmpuşel- Izvoarele Cernei, zonă de contact dintre parcurile naţionale “Retezat” şi “Domogled - Valea Cernei”. Bul. inf. Soc. lepid. rom., 20 (3-4), 119-128
58. Crişan, C. D., Battes, K. P., & Cîmpean, M. 2016 First record of *Bryocamptus (Bryocamptus) mrazeki* (Minkiewicz, 1916) in the Romanian harpacticoid fauna (Copepoda, Harpacticoida). Studia Univ. Babes-Bolyai Biol, 61: 205-212
59. Cupșa, D., Banyai, G. 2006 Contributions on the study of the macrozoobenthic invertebrates from the glacial lakes of the Lăpușnicu Mare River basin (Retezat Mountains, Romania). Transylvanian Review of Systematical and Ecological Research 3: 101-116
60. Curtean-Bănăduc, A., Ciubuc, F., Ciubuc, C. 2006. Caddisfly (Insecta: Trichoptera) larvae communities of Bărbat River basin (Retezat Mountains, Romania). Transylvanian Review of Systematical and Ecological Research 3(3): 89-100
61. Cuvelier, S., & Dincă, V. 2007 New data regarding the butterflies (Lepidoptera: Rhopalocera) of Romania, with additional comments (general distribution in Romania, habitat preferences, threats and protection) for ten localized Romanian species. Phegea, 35(3), 93-115.
62. Damian-Georgescu, A. 1963 Crustacea. Copepoda. Fam. Cyclopidae (forme de apa dulce). Fauna RPR 4, fasc. 6: Editura RPR
63. Damian-Georgescu, A. 1966 Crustacea. Copepoda. Calanoida (forme de apa dulce). Fauna RSR 4, fasc. 8: Editura RSR
64. Damian-Georgescu, A. 1970 Copepoda. Harpacticoida (forme de apa dulce). Fauna RSR 4, fasc. 11: Editura RSR
65. Damian-Georgescu, A. 1976 Observatii asupra Ceratopogonidelor capturate in Parcul national Retezat. Sargetia 12: 285-296
66. Damian-Georgescu, A. 2000 Insecta. Diptera. Fam. Ceratopogonidae. Genul *Culicoides*. Faura Romaniei 11, fasc. 14: Editura Academiei Romane
67. Davideanu, A., Davideanu, G., Ilie, D., Kiss, B. 2004 Family Gerridae Leach 1807 (Insecta, Heteroptera), in Romania. Scientific Annals of the Danube Delta Institute for Research and Development, Tulcea 10: 3-14
68. Decei, P. 1981 Lacurile de munte. Drumetie si pescuit. Editura Sport Turism
69. Decou, V. 1962 Revision der Arten der Gattung Duvalius aus dem Hohlen Rumaniens. Annales Historia Naturalis Museum Hungarium 54: 259-267
70. Demeter, L., Mori, C. 2004 Spatial distribution and habitat characteristics of *Chirocephalus diaphanus* (Branchiopoda: Anostraca) in the Retezat National Park (Southern Carpathians, Romania). Biota 5(1-2): 11-23
71. Dincă, V., Cuvelier, S., Zakharov, E. V., Hebert, P. D., & Vila, R. 2010 Biogeography, ecology and conservation of *Erebia oeme* (Hübner) in the Carpathians (Lepidoptera: Nymphalidae: Satyrinae). Annales de la Société entomologique de France 46 (3-4): 486-498.
72. Dinulescu, G. 1958 Diptera. Familia Tabanidae. Fauna RPR 11, fasc. 2: Editura Academiei RPR
73. Dinulescu, G. 1966 Insecta. Diptera. Fam. Simuliidae. Rauna RSR 11, fasc. 8: Editura Academiei RSR
74. Diószeghy L. 1930 Die Lepidopterenfauna des Retyezatgebirges [The Lepidoptera fauna of the Retezat Mountains]. -- Verhandlungen und Mitteilungen des siebenbürgischen Vereins für Naturwissenschaften zu Hermannstadt 79–80 (1929–1930): 189–289.
75. Diószeghy L. 1935 Die Lepidopterenfauna des Retyezatgebirges. Nachtrag I [The Lepidoptera fauna of the Retezat Mountains. Addendum I]. -- Verhandlungen und Mitteilungen des siebenbürgischen Vereins für Naturwissenschaften zu Hermannstadt 83–84 (1933– 1934): 107–126.
76. Dobre, A. 2000 Contributions to the study of ceratopogonids (Diptera) in Romania. Proceedings of the Institute of Biology 3: 213-215
77. Dobre, A. 2004 Spider fauna from sterile deposits (Retezat Massif-Romania). Travaux Musee d'Histoire Naturelle "Grigore Antipa" 47: 359-365
78. Dobreanu, E., Manolache, C. 1948 Les amphipodes des lacs du Massif Retezat. Notationes Biologicae 6: 95-97
79. Dumitrache, I., Sevianu, E. 2010 Contribuții la cunoașterea faunei de micromamifere din Parcul Național Retezat, cu referire la statutul lor de protecție. Ocrotirea naturii 46: 104-111
80. Dunareanu, I. 1956 Despre prezenta vulturului barbos in Retezat. Probleme de Geografie 3: 294-295
81. Dusa, L. 1970 Contributii la studiul foridelor (Diptera, Phoridae) din Romania. Studia Universitaria Babes-Bolyai, Biologia 15: 113-116
82. Fabritius, K. 1975 Proctotrupoide (Hymenoptera) noi pentru fauna Romaniei. Muzeul Bruckental-Studii si Comunicari, St. nat. 19: 231-234
83. Falca, M. 1993 Numerical structure of Collembola populations from the soils of Retezat national Park (the Carpathians). Revue Roumaine de Biologie - Biologie Animale 38: 13-20
84. Falca, M., Simeanu, I. 1972 Abundenta si distributia pe verticala a lumbricidelor din Rezervatia stiintifica a Parcului National Retezat. Studii si Cercetari de Biologie - Seria Zoologie 24: 467-477
85. Feider, Z. 1955 Arachnida. Acarina. Trombidoidea. Fauna RPR 5, fasc. 1: Editura Academiei RPR
86. Feider, Z., Calugar, M., Vasiliu, N. 1972 Contributie la cunoasterea Oribatidelor (Oribatei) din Parcul National Retezat si din depresiunea Jiului de Vest. Ocrotirea Naturii 16: 5-12
87. Fetykó, K., Urák, I. 2004 A new genus and new species in the Romanian spider fauna (Arachnida: Araneae) from the Gura Zlata (Retezat National Park, Romania). Travaux du Muséum National d’Histoire Naturelle “Grigore Antipa 46: 7-13
88. Fuhn, I. 1967 Arahnide din Parcul National Retezat. Ocrotirea naturii si a mediului inconjurator 11: 97-98
89. Fuhn, I. 1974 Cercetari sinecologice cantitative asupra epigaionului Fetei Retezatului. Sargetia 10: 137-155
90. Fuhn, I., Gherasim, V.F. 1995 Arachnida. Fam. Salticidae. Fauna RSR. 5, fasc. 5: Editura Academiei RSR
91. Fuhn, I., Niculescu-Burlacu, F. 1971 Arachnida. Fam. Lycosidae. Fauna RSR 5, fasc. 3: Editura Academiei RSR
92. Fuhn, I., Oltean, C. 1970 Lista Araneelor din R.S. Romania. Muzeul de Stiintele Naturii Bacău, Studii si Comunicari: 157-196
93. Geacu, S., & Dumitraşcu, M. 2017 Alpine marmot populations after four decades of living in the glacial areas of the Făgăraş, Rodna and Retezat Mountains, Romania. Journal of Environmental Biology 38: 703
94. Georgescu, A. 1984 Ecological estimations on the Gamasidae fauna in the soil of two stationaries in the Retezat Mountains. Recherches ecologiques dans le Parc National de Retezat: 201-208 Academia R.S.R., Cluj Napoca
95. Georgescu, A., Vicol, A. 1988 Cercetari asupra faunei de acarieni edafici din trei ecosisteme forestiere din Parcul National Retezat (Carpatii Sudici). Studii si cercetari de biologie. Seria Biologie Animala 40: 77-82
96. Gheorghiu, V. 1983 Contributie la cunoasterea Tefritidelor (Diptera, Tephritidae) din Parcul national Retezat. Ocrotirea naturii si a mediului inconjurator 27: 49
97. Gheorghiu, V. 1985 Contribution a la connaissance des Tephritides (Diptera, Tephritidae) du Parc national de Retezat (I). Travaux Musee d'Histoire Naturelle "Grigore Antipa" 26: 113-120
98. Gheorghiu, V. 1986 Contribution a la connaisssance des Tephritides (Diptera, Tephritidae) du Parc National de Retezat (II). Travaux Musee d'Histoire Naturelle "Grigore Antipa" 28: 113-120
99. Ghete, S. 1983 Specii de coleoptere cavernicole (fam. Trechidae si Catopidae, ord. Coleoptera) aflate in colectia entomologica a Muzeului Judetean din Deva. Sargetia 13: 149-156
100. Ghira, I et al. 2002 Mapping of Transylvanian herpetofauna. Nymphaea 29: 145-201
101. Ghira, I., Nutu, A. 1986 Prezenta unui juvenil de *Stercorarius* in Parcul National Retezat. Ocrotirea naturii si a mediului inconjurator 30: 50
102. Girlea, D. 1977 Lăstunul de stânca (*Apus melba melba* L.) in Retezatul Mic. Ocrotirea naturii si a mediului inconjurator 21: 43-44
103. Giurginca, A., Plăiaşu, R., Munteanu, C. M. 2007 On some Oniscidea and Diplopoda from the Retezat Massif. First record of *Porcellium productum* Frankenberger, 1940 and *Porcellium recurvatum* Verhoeff, 1901 in Romania. Archives of Biological Sciences 59 (3): 233-238
104. Godeanu, S. 1974 Contributii la cunoasterea sinecologica a zoocenozelor tinovului Gemenele din Parcul national Retezat. Sargetia 10: 155-176
105. Godeanu, S. 1981 Fauna de Testacee din circul Glaciar Bucura (Muntii Retezat). Ocrotirea naturii si a mediului inconjurator 25: 191-200
106. Gozmány L. 1953 New data to the Microlepidopta fauna of the Retyezát range. - Annales Historico-naturales Musei Nationalis Hungarici 45 (series nova 4): 181–185.
107. Gozmány L. 1957 Notes on the generic group *Stomopteryx* Hein., and the descriptions of some new Microlepidoptera. Acta Zoologica Academiae Scientiarum Hungaricae 3 (1–2): 107–135
108. Grossu, A.V. 1981 Gastropoda Romaniae. Vol. 3: Subclasa Pulmonata, Ord. Stylommatophora: Suprafam. Clausiliacea; Achatinacea. Editura Litera, Bucuresti
109. Grossu, A.V. 1983 Gastropoda Romaniae. Ordinul Stylommatophora. 4: Editura Litera, Bucureşti
110. Grossu, A.V. 1986 Gastropoda Romaniae. 1: Editura Litera, Bucureşti
111. Grossu, A.V. 1987 Gastropoda Romaniae. Subclasa Pulmonata. 2: Editura Litera, Bucureşti
112. Grossu, A.V. 1993 The catalogue of the molluscs from Romania. Travaux Musee d'Histoire Naturelle "Grigore Antipa" 33: 291-366
113. Grossu, A.V. 1999 A scientifical collection of Romanian molluscs (shells only) - Prof. Dr. A.V. Grossu's donation to "Grigore Antipa" National Museum of Natural History from Bucharest. Travaux Musee d'Histoire Naturelle "Grigore Antipa" 41: 7-35
114. Harsia, T. 1989 Contributii la cunoasterea colembolelor (Insecta, Apterygota) din jnepenisuri si un molidis de limita din Retezatul Mic. A IV Conferinta de Ecologie, Piatra Neamt: 143 Institutul Central de Biologie
115. Harsia, T. 1992 Structura comunitatilor de colembole (Insecta, Colembola). Parcul National Retezat -Studii Ecologice: 243-247 West Side Computers, Brasov
116. Hut, M. 1992. Structura comunitatilor de uropodide (Acarina-Anactinotrichida). Parcul National Retezat -Studii Ecologice: 237-242 West Side Computers, Brasov
117. Iacob, M. 1975 *Rhagio strigosus* Mg. (Diptera, Rhagionidae)- specie noua pentru fauna Romaniei. Muzeul Bruckental-Studii si Comunicari, St. nat. 19: 219-223
118. Iacob, M. 1985 Contributions a la connaissance de la repartition de quelques representants des familles des Bibionidae et des Rhagionidae (Diptera) dans la R. S. Roumanie. Travaux Musee d'Histoire Naturelle "Grigore Antipa" 27: 187-191
119. Iliuț, I., Pârvulescu, L., Petrovici, M. 2006 The diversity of Tricladids fauna (Platylminthes, Turbellaria) from Retezat National Park (Romania). Transylvanian Review of Systematical and Ecological Research 3: 61-64
120. Ionescu, M.A., Weinberg, M. 1971 Insecta. Diptera-Asilidae. Fauna RSR. 11, fasc. 11: Editura Academiei RSR
121. Iuga, V.G. 1958 Hymenoptera Apoidea. Fam. Apidae. Subfam. Anthophorinae. Fauna RPR 9, fasc. 3: Editura Academiei RPR
122. Kis, B. 1960 Revision der in Rumanien Vorkommended Isophya-arten (Orthoptera, Phaneropterinae). Acta Zoologica 6: 349-369
123. Kis, B. 1984. Les orthopteres du Parc national de Retezat. Recherches ecologiques dans le Parc National de Retezat: 220-223 Academia R.S.R., Cluj Napoca
124. Kis, B. 1997a. Ortopterele din parcurile nationale Retezat si Valea Cernei (Insecta: Orthoptera). Entomofauna parcurilor nationale Retezat si Valea Cernei: 35-41. Societatea Lepidopterologica Romana, Cluj Napoca
125. Kis, B. 1997b. Heteroptere colectate din parcurile nationale Retezat si Valea Cernei. Entomofauna parcurilor nationale Retezat si Valea Cernei: 43-49. Societatea Lepidopterologica Romana, Cluj Napoca
126. Kis, B. 1997c. Plecopterele din parcurile nationale Retezat si Valea Cernei-Domogled (Insecta: Plecoptera). Entomofauna parcurilor nationale Retezat si Valea Cernei: 29-33. Societatea Lepidopterologica Romana, Cluj Napoca
127. Kis, B., Nagler, C., Mandru, C. 1970. Neuroptera (Planipennia). Fauna RSR 8, fasc. 6: Editura RSR
128. Knechtel, W.K. 1955. Insecta. Hymenoptera. Subfam. Apinae. Fauna R.P.R. 9, fasc. 1: Editura Academiei R.P.R.
129. Konig, F. 1969. Cercetari asupra lepidopterelor din Parcul national Retezat. Ocrotirea naturii si a mediului inconjurator 13: 175-181
130. Konig, F. 1983. Contributii la cunoasterea faunei de lepidoptere a judetului Hunedoara. Sargetia 13: 135-144
131. Korponai, J., Magyari, E. K., Buczkó, K., Iepure, S., Namiotko, T., Czakó, D., Kövér, C., Braun, M. 2011. Cladocera response to Late Glacial to Early Holocene climate change in a South Carpathian mountain lake. Hydrobiologia 676(1): 223-235
132. Kovács, S., Kovács, Z. 1992a. *Pyrgus armoricanus* (Lepidoptera, Herperiidae), une espece peu connue en Roumanie. Travaux Musee d'Histoire Naturelle "Grigore Antipa" 32: 77-83
133. Kovács, S., Kovács, Z. 1992b. Contributions a la connaissance de la faune de Larentiinae (Lepidoptera, Geometridae) de Roumanie. Travaux Musee d'Histoire Naturelle "Grigore Antipa" 32: 85-93
134. Kovács, Z., Kovács, S. 1991. Especes de Laspeyresiini (Lepidoptera, Tortricidae) nouvelles pour la faune de Roumanie. Travaux Musee d'Histoire Naturelle "Grigore Antipa" 31: 117-122
135. Kovács, Z., Kovács, S. 1998. Familia Micropterigidae (Lepidoptera) in Romania. Bul. Inf. Soc. Lepid. Rom 9: 29-44.
136. Kovács, Z., & Kovács, S. 2022. An overview of the Romanian Gelechiidae (Lepidoptera) summarizing the current knowledge in an updated and annotated checklist. Entomologica romanica, 26, 1-76.
137. Kovács, Z., & Kovács, S. 2023. An overview of the Romanian Meessiidae, Eriocottidae and Tineidae (Lepidoptera, Tineoidea) summarizing the current knowledge in an updated and annotated checklist. Entomologica romanica, 27, 1-34.
138. Lăcătuşu, M., Filipescu, C. 1989. Hymenoptera. Familia Braconidae. Fauna RSR 9, fasc. 11: Editura Academiei RSR
139. Lehrer, A. 1972. Diptera. Familia Calliphoridae. Fauna RSR 11, fasc. 12: Editura RSR
140. Lehrer, A. 1973. Diptere Sarcophagidae malacofage si lombricidofage din R.S.Romania. Muzeul Judetean Suceava. Studii si Comunicari, Stiintele Naturii 3: 409-435
141. Lehrer, A. 1993. Tabanidae (Diptera) din judetul Hunedoara si unele aspecte arealografice ale acestora in Romania. Sargetia 14-15: 303-330
142. Mack-Fira, V., Onciu, T. 1973. Turbelariate din lacurile glaciare din Carpatii româneşti (Masivul Retezat). Studii si cercetari de biologie. Seria Zoologie 25: 303-308
143. Matic, Z 1985. Note sur les chilopodes du genre *Strigamia* (Chilopoda: Geophilomorpha) de la reserve scientifique de "Retezat". Revue Roumaine de Biologie - Biologie Animale 30: 11-15
144. Matic, Z 1992. Chilopodele din Parcul national Retezat. Parcul National Retezat -Studii Ecologice: 248-250. West Side Computers, Brasov
145. Matic, Z. 1966. Clasa Chilopoda. Subclasa Anamorpha. Fauna RSR. 6, fasc. 1: Editura Academiei RSR
146. Matic, Z. 1972. Chilopoda. Subclasa Epimorpha. Fauna RSR 6, fasc. 2: Editura Academiei RSR
147. Matic, Z., Darabantu, C. 1968. Contributii la cunoasterea genurilor *Strigamia, Pachymerium* si *Dicellophilus* (Chilopoda, Epimorfa) din fauna Romaniei. Studia Unviersitaria Babes-Bolyai, Biologia 13: 91-98
148. Moruzi, C., Balinschi, I. 1967. Cercetari asupra Actinomycetelor din mîlul cîtorva lacuri glaciare. Analele Universitatii Bucuresti. Seria Stiinte Sociale. Biologie 16: 97-104
149. Munteanu, D. 1986. Cercetari asupra populatiilor de păsări (Aves) din Muntii Retezat (I). Studii si cercetari de biologie. Seria Biologie Animala 38: 87-90
150. Munteanu, D. 1987. Cercetari asupra populatiilor de păsări (Aves) din Muntii Retezat (II). Studii si cercetari de biologie. Seria Biologie Animala 39: 171-174
151. Munteanu, D. 1992. Structura specifica a comunitatii de pasari si mamifere din Parcul National Retezat. Parcul National Retezat -Studii Ecologice: 192-199 West Side Computers, Brasov
152. Murariu D., Benedek, A. M. 2005. New Reports on the presence of *Sorex alpinus* Schinz, 1837 (Insectivora: Soricidae) in the Southern Carpathians (Romania). Travaux Musee d'Histoire Naturelle "Grigore Antipa" 48: 395–405
153. Murariu, D. 2000. Mammalia. Insectivora. Rauna Romaniei 16, fasc. 1: Editura Academiei Romane
154. Negrea, A. 1996. Contributie la cunoasterea gasteropodelor endemice din zonele carstice din Romania. Travaux Musee d'Histoire Naturelle "Grigore Antipa" 36: 217-237
155. Negrea, S. 1983. Crustacea. Cladocera. Fauna R.S.R. 4, fasc.12: Editura Academiei RSR
156. Nitzu, E. 1995. *Bembidion (Bembidionetolitzhya) paracomplanatum* (Coleoptera, Trechidae) a new species from the Southern Carpathians (Romania). Travaux Musee d'Histoire Naturelle "Grigore Antipa" 35: 153-160
157. Novak, J., Harvey, M. 2015. The identity of pseudoscorpions of the genus *Diplotemnus* (Pseudoscorpiones: Atemnidae) from Europe and Asia. North-Western Journal of Zoology 11: 316-323
158. Novak, J., Jablonski, D., Christophoryova, J. 2019. Contribution to the pseudoscorpion fauna of Transylvania and the Eastern and Southern Carpathians, Romania (Arachnida: Pseudoscorpiones). North-Western Journal of Zoology 15: 127-134
159. Obraztsov, N.S. 1961 Die Gattungen der Palaearktischen Tortricidae. II. Die Unterfamilie Olethreutinae. 4. Teil, Tijdschrift voor Entomologie. 104: 51—70
160. Onciu, T.M., Radu, A. 2006. Retezat Mountains (Romania) glacial lakes zooplankton biodiversity. Transylvanian Review of Systematical and Ecological Research 3: 49-60
161. Panin, S. 1952. Insecta. Coleoptera. Fam. Cicindelidae. Fauna R.P.R. 10, fasc. 1: Editura Academiei R.P.R.
162. Panin, S. 1955a. Insecta. Coleoptera. Fam. Carabaidae. Fauna R.P.R. 10, fasc. 2: Editura Academiei R.P.R.
163. Panin, S. 1955b. Insecta. Coleoptera. Fam. Scarabaeidae. Fauna R.P.R. 10, fasc. 3: Editura Academiei R.P.R.
164. Panin, S. 1957. Coleoptera. Fam. Scarabaeidae. Fauna RPR 10, fasc. 4: Editura Academiei RPR
165. Panin, S., Savulescu, N. 1961. Insecta. Coleoptera. Fam. Cerambycidae. Fauna RPR 10, fasc.5: Editura Academiei RPR
166. Paraschivescu, D. 1975. Cercetari asupra Formicidelor apartinind colectiilor Dr. A. Muller si Dr. E. Worell din patrimoniul Muzeului de Istorie Naturala Sibiu. Muzeul Bruckental-Studii si Comunicari, St. nat. 19: 237-246
167. Paraschivescu, D. 1976. Cercetari ecologice asupra formicidelor din Masivele Bucegi si Retezat. Muzeul Bruckental-Studii si Comunicari, St. nat. 20: 193-208
168. Parvu, C. 1980. Contributie la cunoasterea Tabanidelor (Diptera) din Parcul national Retezat. Ocrotirea naturii si a mediului inconjurator 24: 179-183
169. Parvu, C. 1981. Data on the occurrence and distribution of some Tabanids (Diptera) in Romania. Travaux Musee d'Histoire Naturelle "Grigore Antipa" 23: 155-162
170. Parvu, C. 1983. Dolichopodidae (Diptera) de la Gura Zlata (Parcul National Retezat). Ocrotirea naturii si a mediului inconjurator 27: 47-48
171. Parvu, C. 1983. Tabanidae (Diptera) din colectiile Muzeului de istorie naturala din Sibiu. Muzeul Bruckental-Studii si Comunicari, St. nat. 25: 303-313
172. Parvu, C. 1985. *Chrysops divaricatus* and *Tabanus (Glaucops) hirsutus* (Diptera, Tabanidae) in Romania's fauna. Travaux Musee d'Histoire Naturelle "Grigore Antipa" 27: 157-163
173. Parvu, C. 1986. Data on some Dolichopodidae (Diptera) of Romania (IV). Travaux Musee d'Histoire Naturelle "Grigore Antipa" 28: 101-111
174. Parvu, C. 1987. Contribution concerning the distribution of family Dolichopodidae (Diptera) in Romania (V), with the description of a new species, *Hercostomus transsylvanicus* n.sp.. Travaux Musee d'Histoire Naturelle "Grigore Antipa" 29: 169-184
175. Parvu, C. 1991. new data on the distribution of family Dolichopodidae (Diptera) in Romania (VII), with the description of *Hercostomus dacicus* n.sp.. Travaux Musee d'Histoire Naturelle "Grigore Antipa" 31: 123-137
176. Parvu, C. 1995. New data on the distribution, systematics and phenology of the flies with long legs (Diptera: Dolichopodidae) from Romania (IX). Travaux Musee d'Histoire Naturelle "Grigore Antipa" 35: 387-406
177. Parvu, C. 1997. Tabanidae, Dolichopodidae, Empidoidea si Chloropidae (Diptera) semnalate din parcurile nationale Retezat si Valea Cernei intre anii 1865-1994, cu o schita preliminara de zoogeografie si elemente de monitoring. Entomofauna parcurilor nationale Retezat si Valea Cernei: 213-228 Societatea Lepidopterologica Romana, Cluj Napoca
178. Parvu, C. 1992. A synthesis on the distribution of Empididae (Diptera) in Romania with the description of a new species of *Hilara*. Travaux Musee d'Histoire Naturelle "Grigore Antipa" 32: 95-120
179. Parvu, C. 1993. Genus *Haematopota* (Diptera, Tabanidae) in Romania. A study about female genitalia, variability and distribution of the species. Travaux Musee d'Histoire Naturelle "Grigore Antipa" 33: 165-199
180. Parvu, C. 1992. New data on the distribution of family Dolichopodidae (Diptera) in Romania (VIII). Travaux Musee d'Histoire Naturelle "Grigore Antipa" 32: 121-129
181. Parvu, C. 1993. The Tabanid species (Diptera, Tabanidae) of Romania, preserved in the scientific collections of "Grigore Antipa" Natural History Museum of Bucharest. Travaux Musee d'Histoire Naturelle "Grigore Antipa" 33: 201-214
182. Pârvulescu, L., Iliuț, I., Petrovici, M. 2006. The diversity of crustacean fauna (Arthropoda, Crustacea) from Retezat National Park (Romania). Transylvanian Review of Systematical and Ecological Research 3: 65-68
183. Pârvulescu, L., Petrescu, I. 2010. The distribution of stone crayfish *Austropotamobius torrentium* (Schrank, 1803) (Crustacea: Decapoda: Astacidae) in the south-west Romanian mountain and sub-mountain area. Travaux Musee d'Histoire Naturelle "Grigore Antipa" 53: 103-111
184. Pascu, M. 1978. Catalogul Himenopterelor (subord. Symphyta, Fam, Xiphydriidae, Siricidae, Cephidae, Argidae, Cimbicidae si Diprionidae) din colectia Muzeului de Istorie Naturala Sibiu - Romania. Muzeul Bruckental-Studii si Comunicari, St. nat. 22: 329-338
185. Pascu, M. 1979. Subfamilia Apinae (Hymenoptera) in colectia Muzeului de Istorie Naturala din Sibiu. Muzeul Bruckental-Studii si Comunicari, St. nat. 23: 309-317
186. Pascu, M. 1982. Familia Megalodontidaesi Pamphiliidae (Hymenoptera, Tenthredinoidea) in colectiile Muzeuluil de Istorie Naturala Sibiu. Muzeul Bruckental-Studii si Comunicari, St. nat. 24: 439-442
187. Pascu, M. 1984. Catalogul fam. Vespidae si Eumenidae (Hymenoptera) din colectia Muzeului de Istorie naturala Sibiu. Muzeul Bruckental-Studii si Comunicari, St. nat. 26: 353-361
188. Pascu, M. 2004. Catalogul suprafamiliei Apoidea (Hymenoptera): Colletidae, Halictidae, Andrenidae, Melittidae, Megachilidae, Anthophoridae si Apidae din colectiile Muzeului de Istorie Naturala din Sibiu - Partea I. Muzeul Bruckenthal-Studii si Comunicari, St. nat. 29: 147-163
189. Pascu, M. 2006. Catalogul suprafamiliei Apoidea (Hymenoptera): Colletidae, Halictidae, Andrenidae, Melittidae, Megachilidae, Anthophoridae si Apidae din colectiile Muzeului de Istorie Naturala din Sibiu - Partea II. Brukenthal. Acta Musei I.3: 99-106
190. Peterfi, F. 1974. Genul *Cetema* (Diptera, Chloropidae) in fauna Romaniei. Studia Unviersitaria Babes-Bolyai, Biologia 19: 118-123
191. Peterfi, L.S. 1974. Flora algala din complexul mlastinos Valea Judele Zănoaga, Parcul national Retezat. Sargetia 10: 85-94
192. Pisica, C. 1977. Specii de Ichneumonidae (Hymenoptera) rare in fauna Romaniei. Anuarul Muzeuluil de Stiinte Naturale Piatra Neamt, seria Botanica-Zoologie 3: 215-218
193. Plattner, H. 1963. Odonate din sudul Transilvaniei. Comunicarile Academiei RPR 11: 969-976
194. Pop, V. V., Pop, A. A., & Csuzdi, C. 2007. An updated viewpoint on the earthworm communities with the *Dendrobaena alpina* species group (Oligochaeta, Lumbricidae) from the South-Eastern Carpathians. European Journal of Soil Biology 43: S53-S56
195. Pop, V.V. 1972. Contributii la studiul lumbricidelor (Oligochaeta) din Parcul National Retezat. Ocrotirea Naturii 16: 33-41
196. Pop, V.V. 1984. Seasonal dynamics of the earthworms in the Retezat National Park. Recherches ecologiques dans le Parc National de Retezat: 185-196. Academia R.S.R., Cluj Napoca
197. Pop, V.V. 1992. Structura si dinamica comunitatilor de lumbricide. Parcul National Retezat -Studii Ecologice: 215-227. West Side Computers, Brasov
198. Popescu-Gorj, A. 1983. Retrospectiva privind cercetarile asupra faunei de lepidoptere a judetului Hunedoara. Sargetia 13: 125-134
199. Popescu-Gorj, A. 1985. Nouvelles donnees pour la connaissance des Microlepidopterea de Roumanie. Travaux Musee d'Histoire Naturelle "Grigore Antipa" 27: 101-119
200. Popescu-Gorj, A.1989. Nouvelles donnees pour la connaissance des Lepidopteres de la faune de Roumanie. Travaux Musee d'Histoire Naturelle "Grigore Antipa" 30: 71-79
201. Popescu-Gorj, A. 1994. *Erebia manto* (Lepidoptera) in the Romanian Carpathians. Travaux Musee d'Histoire Naturelle "Grigore Antipa" 34: 69-79
202. Popescu-Gorj, A. 1986. Especes nouvelles ou peu connues dans la faune de microlepidopteres de Roumanie. Travaux Musee d'Histoire Naturelle "Grigore Antipa" 28: 79-83
203. Popescu-Gorj, A. 1986. *Erebia pharte* Hbn. (Lepidoptera, Satyridae), a species less known in the Romanian Carpathians. Travaux Musee d'Histoire Naturelle "Grigore Antipa" 28: 8588
204. Popescu-Gorj, A., Niculescu, E., Alexinschi, A. 1958. Lepidoptera. Familia Aegeriidae. Fauna RPR 11, fasc. 1: Editura Academiei RPR
205. Popovici, I. 1992. Structura si dinamica comunitatilor de nematode (Nematoda). Parcul National Retezat -Studii Ecologice: 200-214. West Side Computers, Brasov
206. Proches, S. 1999. Catalogue of the superfamily Curculionoidea (Coleoptera) - "Nicolae Savulescu" collection at the "Grigore Antipa" National Museum of Natural History (Bucharest). Travaux Musee d'Histoire Naturelle "Grigore Antipa" 41: 315-324
207. Prunescu-Arion, E., Toniuc, N. 1967. Contributii la studiul lacurilor alpine Gemenele si Taul Negru din Parcul National Retezat. Ocrotirea naturii si a mediului inconjurator 11: 219-223
208. Purice, D.M. 2000. Aspects concerning the dynamics of carabid beetles fauna from some mountain riparian ecosystems. Proc. Inst. Biol. 3: 117-125
209. Purice, D.M. 2002. Aspects on the structure of the carabid beetles communities (ord. Coleoptera, fam. Carabidae) in some forest ecosystems of the Retezat Mountains. Proc. Inst. Biol. 4: 157-164
210. Radu, G.V. 1983. Crustacea. Ordinul Isopoda. Subord. Oniscoidea. Oniscoidee inferioare. Fauna R.S.R. 4, fasc. 13: Editura Academiei R.S.R.
211. Radu, G.V. 1985. Crustacea. Ordinul Isopoda.Subord. Oniscoidea. Tribul Crinochaeta. Fauna R.S.R. 4, fasc. 14: Editura Academiei R.S.R.
212. Rafinski, J., Cogalniceanu, D., Babik, W. 2001. Genetic differentiation of the two subspecies of the smooth newt inhabiting Romania, *Triturus vulgaris vulgaris* and *T. v. ampelensis* (Urodela, Salamandridae) as revealed by enzyme electrophoresis. Folia biologica (Krakow) 49: 239-245
213. Raianu, L. 1970. Catalogul speciilor de *Philonthus* (Staphilinidae) din colectiile Muzeului de Istorie Naturala din Sibiu. Muzeul Bruckental-Studii si Comunicari, St. nat. 15: 287-306
214. Rakosy, L. 1992. Macrolepidopterele din Parcul National Retezat. Parcul National Retezat -Studii Ecologice: 254-282. West Side Computers, Brasov
215. Rakosy, L. 1997. Macrolepidopterele din Parcul National Retezat. Entomofauna parcurilor nationale Retezat si Valea Cernei: 87-122Societatea Lepidopterologica Romana, Cluj Napoca
216. Rudescu, L. 1960. Trochelminthes. Rotatoria. Fauna RPR 2, fasc. 2: Editura Academiei RPR
217. Ruicanescu, A. 1993. Contributii la studiul faunei de Dytiscoidea si Gyrinoidea (Insecta, Coleoptera) din Sud-Vestul Transilvaniei. Sargetia 14-15: 125-129
218. Ruicanescu, A. 1997. Coleoptere colectate din parcurile nationale Retezat si Valea Cernei. Entomofauna parcurilor nationale Retezat si Valea Cernei: 57-73. Societatea Lepidopterologica Romana, Cluj Napoca
219. Rusti, D. 1994. Additional data to the checklist of Romanian lepidoptera (Insecta, Lepidoptera). Travaux Musee d'Histoire Naturelle "Grigore Antipa" 34: 81-93
220. Ruzicka, V 1985. *Lepthyphantes retezaticus* sp. n., a new spider from the Rumanian Mountains (Araneae, Linyphiidae). Vest. cs. Spolec. zool. 49: 234-238
221. Schneider, E. 1975. Genul *Amara* Bonelli in colectiile Muzeului de Istorie Naturala din Sibiu (Coleoptera, Carabidae). Muzeul Bruckental-Studii si Comunicari, St. nat. 19: 185-196
222. Scobiola-Palade, X.G. 1978. Insecta. Hymenoptera. Symphyta. Tenthredinoidea. Fauna RSR. 9 fasc. 8: Editura Academiei RSR
223. Scobiola-Palade, X.G. 1981. Hymenoptera. Symphyta. Tenthredinoidea. Fauna RSR 9, fasc. 9: Editura RSR
224. Serafim, R. 1991. Les Coccinellides (Coleoptera) gardes dans la collection du Museum d'Histoire Naturelle "Grigore Antipa" (Bucarest). Travaux Musee d'Histoire Naturelle "Grigore Antipa" 31: 223-253
225. Serafim, R. 1994. Donnes sur la tribu Scymnini (Coleoptera, Coccinellidae) en Roumanie. Travaux Musee d'Histoire Naturelle "Grigore Antipa" 34: 95-115
226. Stahlavsky, F., Christophoryova, J., Henderickx, H. 2013. A karyological study of four European species of *Roncus* (Pseudoscopiones: Neobisiidae). Eur. J. Entomol. 110: 393-399
227. Stănescu, A. 2001. Catalogue of Mirids (Heteroptera: Miridae) from Romania. Travaux Musee d'Histoire Naturelle "Grigore Antipa" 43: 129-197
228. Stănescu, C. 1982. Date privind genul *Rhingia* Scopoli 1763 (Diptera, Syrphidae) din Romania. Muzeul Bruckental-Studii si Comunicari, St. nat. 24: 413-419
229. Stănescu, C. 1983. Catalogul speciilor de Sirfide (Diptera, Syrphidae) din colectiile Muzeului de Istorie naturala din Sibiu. Muzeul Bruckental-Studii si Comunicari, St. nat. 25: 315-345
230. Stănescu, C. 1992. Sur le genre *Paragus* (Diptera, Syrphidae) en Roumanie. Travaux Musee d'Histoire Naturelle "Grigore Antipa" 32: 197-209
231. Stănescu, M. 1995. The catalogue of "Ludovic Beregszaszy" Lepidopteran collection (Insecta: Lepidoptera). Travaux Musee d'Histoire Naturelle "Grigore Antipa" 35: 221-346
232. Stelian, R. 1999. Retezat National Park and Biosphere Reserve. The National Parks and Biosphere Reserves in Carpathians. The Last Nature Paradises: 234-244 WWF/IUCN/UNESCO MAB
233. Sterghiu, C. 1985. Arachnida. Fam. Clubionidae. Fauna RSR. 5, fasc. 4: Editura Academiei RSR
234. Stugren, B., Ghira, I. 1992. Cercetari faunistice si de bioproductivitate asupra amfibienilor si reptilelor din Muntii Retezat. Parcul National Retezat -Studii Ecologice: 189-191. West Side Computers, Brasov
235. Suciu, M., Aioanei, F. 1991. Taxonomic and zoogeographic data on genus *Rhadinopsylla* (Syphonaptera, Hystrichopsyllidae) in Romania. Travaux Musee d'Histoire Naturelle "Grigore Antipa" 31: 265-284
236. Tabacaru, I., Giurginca, A., Vanoaia, L. 2003. Cavernicolous Diplopoda of Romania. Trav. Inst. Speol. "Emil Racovitza" 41-42: 121-148
237. Tatole, V. 1994. Impactul haldelor de steril asupra faunei reofile (nevertebrate) din bazinul Raul Mare (Masivul Retezat). Ocrotirea naturii si a mediului inconjurator 38: 115-122
238. Tatole, V. 1996. O noua contributie la cunoasterea chironomidofaunei Romaniei: G. *Limnophyes* (Diptera, Chironomidae). Studii si cercetari de biologie, serie Biologie Animala 48: 105-112
239. Teodor L.A., Milin, V.Ș. 2014. Weevils (Coleoptera: Curculionidea) from the southern part of Retezat National Park and the Domogled-Cerna Valley National Park, Romania. Buletinul informativ al Societăţii Lepidopterologice Române 24: 44-57
240. Teodoreanu, M. 1970. Contributii la cunoasterea raspindirii genului *Pterostichus* (Coleoptera, Carabidae) in Romania. Studia Unviersitaria Babes-Bolyai, Biologia 15: 117-122
241. Teodoreanu, M. 1984. Preliminary research on the edaphic Coleoptera in two timberline forest ecosystems in the Retezat Mountains. Recherches ecologiques dans le Parc National de Retezat : 224-230. Academia R.S.R., Cluj Napoca
242. Tittizer, T.G. 1968. Hydrobiologische untersuchungen im Zanoaga-See es Retezat-Massives. Travaux Musee d'Histoire Naturelle "Grigore Antipa" 7: 183-197
243. Tomescu, R. 1984. Specific structure of Protozoa in forest ecosystems in the Retezat Massif (Romania). Recherches ecologiques dans le Parc National de Retezat : 170-174. Academia R.S.R., Cluj Napoca
244. Ujvarosi, L. 1997. Fauna de Trichoptere din Masivul Retezat si Valea Cernei (Insecta: Trichoptera). Entomofauna parcurilor nationale Retezat si Valea Cernei: 75-86 Societatea Lepidopterologica Romana, Cluj Napoca
245. Ujvarosi, L. 2003. Records of new and insufficiently known species of crane flies (Diptera: Tipulidae) in Romania. Entomol. Rom. 7: 51-62
246. Ujvarosi, L., Stary, J. 2003. A new *Pedicia* (*Crunobia*) from Romania and other four species new to the country's fauna (Diptera: Pediciidae). Entomol. Rom. 7: 45-50
247. Urák, I. 2001. Contribuţii la cunoaşterea faunei de păianjeni (Arachnida: Araneae) din Rezervaţia Biosferei Parcul Naţional Retezat. Buletinul informativ al Societăţii Lepidopterologice Române 12(1-4): 241-250
248. Urák, I., Fetykó, K. 2006. Arachnological studies in the Retezat National Park (Romania). Transylvanian Review of Systematical and Ecological Research 3: 79-88
249. Valenciuc, N. 2002. Mammalia. Chiroptera. Fauna Romaniei 16, fasc. 3: Editura Academiei Romane
250. Vasiliu, G.D.1948. Quelques indications sur la faune ichtyologique des lacs du Massif Retezat. Notationes Biologicae 6: 89-92
251. Vasiliu, N.1992. Structura biocenotica a comunitatilor de oribatide (Acarina-Oribatei) din Masivul Retezat. Parcul National Retezat -Studii Ecologice: 228-236. West Side Computers, Brasov
252. Vizauer, T. C. 2001. Contribuţia publicaţiilor entomologice din Ungaria la cunoaşterea faunei de lepidoptere din Transilvania. I. Publicaţii din revista „Rovartani Lapok”. Buletinul informativ al Societăţii Lepidopterologice Române 11(1-4): 85-97
253. Weinberg, M. 1970. Familia Conopidae (Diptera) din colectiile Muzeului Bruckenthal. Muzeul Bruckenthal-Studii si Comunicari, St. nat. 15: 273-278
254. Weinberg, M. 1985. New data on the presence and distribution of the Therevidae (Diptera) in Romania. Travaux Musee d'Histoire Naturelle "Grigore Antipa" 27: 129-149
255. Weinberg, M. 1987. Species of Pipunculidae (Diptera) mentioned for the first time in the fauna of Romania. Travaux Musee d'Histoire Naturelle "Grigore Antipa" 29: 165-167
256. Weinberg, M. 1996. Nota cu privire la prezenta Asilidelor (Diptera, Asilidae) in Parcul National Retezat (Romania). Travaux Musee d'Histoire Naturelle "Grigore Antipa" 36: 251-253
