# Supplementary material for: Awaiting discovery: How biases in faunistic surveys hinder conservation in mountain protected areas—A case study from Romania’s oldest national park
Source: PLoS One. 2025 Apr 1;20(4):e0319871. doi: 10.1371/journal.pone.0319871 (PMC11961002; doi:10.1371/journal.pone.0319871)
Supplement: S3 Table — The table presents a summary of the animal diversity reported from RNP, according to the scientific literature and GBIF [35]. The taxonomy follows GBIF Backbone Taxonomy [4] and the Catalogue of Life (COL) [3]. NA = “not assigned”. Taxa are ordered alphabetically. The numbers in parentheses represent the total number of unique items of each category. (DOCX) [file pone.0319871.s003.docx]

**S3 Table.** Synthetic overview of the faunistic diversity reported from Retezat National Park (RNP), based on data from the scientific literature and GBIF. The table presents a summary of the animal diversity reported from RNP, according to the scientific literature and GBIF [35]. The taxonomy follows GBIF Backbone Taxonomy [4] and the Catalogue of Life (COL) [3]. NA = “not assigned”. Taxa are ordered alphabetically. The numbers in parentheses represent the total number of unique items of each category.

| **Phylum (9)** | **Class (28)** | **Order (99)** | **Families (494)** | **Species (4374)** | **Genera (2113)** | **Papers (256)** | **Species in GBIF only (73)** |
| --- | --- | --- | --- | --- | --- | --- | --- |
| Annelida | Clitellata | Crassiclitellata | 1 | 16 | 7 | 4 |  |
| Annelida | Clitellata | Enchytraeida | 1 | 42 | 8 | 2 |  |
| Annelida | Clitellata | Haplotaxida | 1 | 1 | 1 | 1 |  |
| Annelida | Clitellata | Lumbriculida | 1 | 1 | 1 | 1 |  |
| Annelida | Clitellata | Rhynchobdellida | 1 | 1 | 1 | 1 |  |
| Annelida | Clitellata | Tubificida | 1 | 4 | 3 | 1 |  |
| Arthropoda | Arachnida | Araneae | 21 | 168 | 91 | 9 | 1 |
| Arthropoda | Arachnida | Mesostigmata | 10 | 32 | 13 | 2 | 1 |
| Arthropoda | Arachnida | Opiliones | 4 | 15 | 12 | 2 |  |
| Arthropoda | Arachnida | Pseudoscorpiones | 3 | 5 | 4 | 4 |  |
| Arthropoda | Arachnida | Sarcoptiformes | 28 | 105 | 59 | 2 | 2 |
| Arthropoda | Arachnida | Trombidiformes | 10 | 27 | 14 | 2 |  |
| Arthropoda | Branchiopoda | Anostraca | 2 | 2 | 2 | 3 |  |
| Arthropoda | Branchiopoda | Diplostraca | 2 | 8 | 4 | 3 |  |
| Arthropoda | Chilopoda | Geophilomorpha | 2 | 14 | 4 | 1 |  |
| Arthropoda | Chilopoda | Lithobiomorpha | 1 | 15 | 3 | 1 |  |
| Arthropoda | Chilopoda | Scolopendromorpha | 1 | 4 | 1 | 1 |  |
| Arthropoda | Collembola | Entomobryomorpha | 5 | 38 | 17 | 3 |  |
| Arthropoda | Collembola | Neelipleona | 1 | 1 | 1 | 1 |  |
| Arthropoda | Collembola | Poduromorpha | 6 | 44 | 22 | 2 |  |
| Arthropoda | Collembola | Symphypleona | 4 | 5 | 5 | 1 |  |
| Arthropoda | Copepoda | Calanoida | 1 | 2 | 2 | 1 |  |
| Arthropoda | Copepoda | Cyclopoida | 1 | 8 | 4 | 4 |  |
| Arthropoda | Copepoda | Harpacticoida | 1 | 7 | 4 | 1 |  |
| Arthropoda | Diplopoda | Chordeumatida | 2 | 3 | 2 | 1 |  |
| Arthropoda | Diplopoda | Glomerida | 1 | 3 | 2 | 3 |  |
| Arthropoda | Diplopoda | Julida | 1 | 16 | 12 | 3 | 1 |
| Arthropoda | Diplopoda | Polydesmida | 2 | 7 | 2 | 3 |  |
| Arthropoda | Diplopoda | Polyzoniida | 1 | 1 | 1 | 1 |  |
| Arthropoda | Insecta | Coleoptera | 24 | 300 | 146 | 25 | 9 |
| Arthropoda | Insecta | Diptera | 32 | 666 | 237 | 51 | 6 |
| Arthropoda | Insecta | Ephemeroptera | 3 | 14 | 6 | 3 |  |
| Arthropoda | Insecta | Hemiptera | 22 | 116 | 88 | 4 | 3 |
| Arthropoda | Insecta | Hymenoptera | 23 | 485 | 220 | 16 |  |
| Arthropoda | Insecta | Lepidoptera | 57 | 1390 | 653 | 21 | 20 |
| Arthropoda | Insecta | Megaloptera | 1 | 1 | 1 | 1 |  |
| Arthropoda | Insecta | Neuroptera | 2 | 4 | 3 | 1 |  |
| Arthropoda | Insecta | Odonata | 3 | 3 | 3 | 2 | 1 |
| Arthropoda | Insecta | Orthoptera | 4 | 50 | 30 | 1 | 2 |
| Arthropoda | Insecta | Plecoptera | 7 | 56 | 19 | 6 | 2 |
| Arthropoda | Insecta | Psocodea | 8 | 23 | 14 | 1 |  |
| Arthropoda | Insecta | Siphonaptera | 1 | 1 | 1 | 1 |  |
| Arthropoda | Insecta | Trichoptera | 15 | 102 | 47 | 8 |  |
| Arthropoda | Malacostraca | Amphipoda | 2 | 3 | 2 | 2 | 1 |
| Arthropoda | Malacostraca | Decapoda | 1 | 1 | 1 | 1 |  |
| Arthropoda | Malacostraca | Isopoda | 4 | 13 | 5 | 4 |  |
| Arthropoda | Ostracoda | Podocopida | 1 | 2 | 2 | 1 |  |
| Chordata | Actinopterygii | Cypriniformes | 3 | 7 | 7 | 3 |  |
| Chordata | Actinopterygii | Salmoniformes | 1 | 5 | 5 | 2 |  |
| Chordata | Actinopterygii | Scorpaeniformes | 1 | 1 | 1 | 1 |  |
| Chordata | Amphibia | Anura | 3 | 5 | 4 | 7 |  |
| Chordata | Amphibia | Caudata | 1 | 3 | 3 | 1 |  |
| Chordata | Aves | Accipitriformes | 1 | 7 | 6 | 2 | 1 |
| Chordata | Aves | Anseriformes | 1 | 3 | 2 | 1 | 1 |
| Chordata | Aves | Apodiformes | 1 | 1 | 1 | 1 |  |
| Chordata | Aves | Charadriiformes | 3 | 4 | 4 | 2 |  |
| Chordata | Aves | Ciconiiformes | 1 | 1 | 1 | 0 | 1 |
| Chordata | Aves | Columbiformes | 1 | 3 | 2 | 1 | 2 |
| Chordata | Aves | Cuculiformes | 1 | 1 | 1 | 1 |  |
| Chordata | Aves | Falconiformes | 1 | 2 | 1 | 1 |  |
| Chordata | Aves | Galliformes | 2 | 5 | 5 | 1 | 2 |
| Chordata | Aves | Gruiformes | 1 | 1 | 1 | 1 |  |
| Chordata | Aves | Passeriformes | 21 | 65 | 46 | 1 | 11 |
| Chordata | Aves | Pelecaniformes | 1 | 2 | 1 | 0 | 2 |
| Chordata | Aves | Piciformes | 1 | 7 | 4 | 1 | 2 |
| Chordata | Aves | Strigiformes | 1 | 4 | 3 | 1 |  |
| Chordata | Mammalia | Artiodactyla | 3 | 4 | 4 | 1 |  |
| Chordata | Mammalia | Carnivora | 4 | 9 | 7 | 1 |  |
| Chordata | Mammalia | Chiroptera | 2 | 5 | 3 | 2 |  |
| Chordata | Mammalia | Erinaceomorpha | 1 | 1 | 1 | 1 |  |
| Chordata | Mammalia | Lagomorpha | 1 | 1 | 1 | 1 |  |
| Chordata | Mammalia | Rodentia | 4 | 17 | 12 | 10 |  |
| Chordata | Mammalia | Soricomorpha | 2 | 8 | 4 | 8 |  |
| Chordata | Petromyzonti | Petromyzontiformes | 1 | 1 | 1 | 1 |  |
| Chordata | Squamata | NA | 6 | 9 | 8 | 7 |  |
| Gastrotricha | NA | Chaetonotida | 2 | 22 | 7 | 1 |  |
| Mollusca | Bivalvia | Sphaeriida | 1 | 3 | 1 | 3 |  |
| Mollusca | Gastropoda | Architaenioglossa | 1 | 3 | 2 | 3 |  |
| Mollusca | Gastropoda | Ellobiida | 1 | 1 | 1 | 1 |  |
| Mollusca | Gastropoda | Littorinimorpha | 1 | 2 | 1 | 2 |  |
| Mollusca | Gastropoda | Stylommatophora | 23 | 92 | 56 | 10 | 2 |
| Nematoda | Chromadorea | Araeolaimida | 2 | 17 | 7 | 1 |  |
| Nematoda | Chromadorea | Monhysterida | 1 | 4 | 3 | 2 |  |
| Nematoda | Chromadorea | Rhabditida | 16 | 53 | 33 | 2 |  |
| Nematoda | Enoplea | Dorylaimida | 7 | 39 | 19 | 2 |  |
| Nematoda | Enoplea | Enoplida | 4 | 13 | 5 | 2 |  |
| Nematoda | Enoplea | Merminthida | 1 | 1 | 1 | 1 |  |
| Nematoda | Enoplea | Mononchida | 4 | 11 | 7 | 2 |  |
| Nematoda | Enoplea | Triplonchida | 5 | 9 | 6 | 1 |  |
| Platyhelminthes | NA | Prorhynchida | 1 | 1 | 1 | 1 |  |
| Platyhelminthes | NA | Rhabdocoela | 3 | 6 | 6 | 2 |  |
| Platyhelminthes | NA | Tricladida | 3 | 16 | 7 | 4 |  |
| Platyhelminthes | NA | Catenulida | 2 | 3 | 3 | 1 |  |
| Rotifera | Eurotatoria | Bdelloidea | 3 | 17 | 5 | 2 |  |
| Rotifera | Eurotatoria | Collothecacea | 1 | 1 | 1 | 1 |  |
| Rotifera | Eurotatoria | Flosculariacea | 1 | 1 | 1 | 1 |  |
| Rotifera | Eurotatoria | Ploima | 11 | 42 | 16 | 4 |  |
| Tardigrada | Eutardigrada | Apochela | 1 | 1 | 1 | 1 |  |
| Tardigrada | Eutardigrada | Parachela | 2 | 6 | 2 | 1 |  |
| Tardigrada | Heterotardigrada | Echiniscoidea | 1 | 3 | 2 | 1 |  |
